# Left-hemisphere glioma drives systematic patterns of contralesional functional connectivity

**DOI:** 10.1093/braincomms/fcaf349

**Published:** 2025-09-11

**Authors:** Emma Strawderman, Frank E Garcea, Madalina E Tivarus, Steven P Meyers, Adnan A Hirad, William M Burns, Kevin A Walter, Tyler Schmidt, Webster H Pilcher, Bradford Z Mahon

**Affiliations:** Department of Neuroscience, University of Rochester Medical Center, Rochester, NY 14642, USA; Department of Neurosurgery, University of Rochester Medical Center, Rochester, NY 14642, USA; Department of Neuroscience, University of Rochester Medical Center, Rochester, NY 14642, USA; Department of Neurosurgery, University of Rochester Medical Center, Rochester, NY 14642, USA; Department of Neuroscience, University of Rochester Medical Center, Rochester, NY 14642, USA; Department of Imaging Sciences, University of Rochester Medical Center, Rochester, NY 14642, USA; Department of Neuroscience, University of Rochester Medical Center, Rochester, NY 14642, USA; Department of Neurosurgery, University of Rochester Medical Center, Rochester, NY 14642, USA; Department of Imaging Sciences, University of Rochester Medical Center, Rochester, NY 14642, USA; Department of Neuroscience, University of Rochester Medical Center, Rochester, NY 14642, USA; Department of Surgery, University of Rochester Medical Center, Rochester, NY 14642, USA; Department of Neurosurgery, University of Rochester Medical Center, Rochester, NY 14642, USA; Department of Neurosurgery, University of Rochester Medical Center, Rochester, NY 14642, USA; Department of Neurosurgery, University of Rochester Medical Center, Rochester, NY 14642, USA; Department of Neurosurgery, University of Rochester Medical Center, Rochester, NY 14642, USA; Department of Neurosurgery, University of Rochester Medical Center, Rochester, NY 14642, USA; Department of Psychology, Carnegie Mellon University, 5000 Forbes Avenue, Pittsburgh, PA 15213, USA; Neuroscience Institute, Carnegie Mellon University, 5000 Forbes Avenue, Pittsburgh, PA 15213, USA

**Keywords:** functional reorganization, resting-state functional connectivity, glioma, machine learning, isocitrate dehydrogenase mutation

## Abstract

Gliomas can cause changes in functional networks both proximal and distal to the lesion. Understanding glioma-induced functional reorganization has implications for understanding variability across patients in cognitive outcomes, disease progression, and survival. Here, we leverage machine learning techniques to show that left-hemisphere gliomas are associated with systematic changes in right-hemisphere connectivity. We analyzed right-hemisphere functional connectivity patterns from resting-state functional MRI in 48 patients with left-hemisphere gliomas (mean age 50 years, 31 males) and 107 neurotypical controls (mean age 49 years, 44 males). We employed machine learning techniques, including support vector machines, to assess whether the pattern of right-hemispheric resting-state functional connectivity could distinguish left-hemisphere glioma patients from controls, and predict glioma characteristics, including isocitrate dehydrogenase mutation, World Health Organization grade, and relative size. A support vector machine binary classifier distinguished patients from controls based on right-hemisphere connectivity with 89% accuracy and 84% precision (both *P* = 0.001), indicating consistent contralesional connectivity differences as a function of glioma. The model also achieved 79% sensitivity for detecting patients (*P* = 0.028). Furthermore, patients with similar right-hemisphere connectivity profiles had lesions in similar locations within the left hemisphere, suggesting that the observed connectivity changes are influenced by glioma location. Additionally, the pattern of right-hemisphere connectivity could predict the presence of left-hemisphere gliomas specifically in regions of the parietal lobe. We also found that distinct contralesional connectivity patterns classified glioma molecular subtypes, achieving 78% accuracy in classifying patients by isocitrate dehydrogenase mutation (*P* = 0.004), with 82% precision (*P* = 0.003) and 73% sensitivity (*P* = 0.048) for mutant-tumors. However, right-hemisphere functional connectivity could not distinguish patients based on their tumor grade or relative size, with models performing no different from chance. These findings provide evidence for systematic changes in the contralesional connectome in glioma patients, consistent with theories of glioma-induced functional reorganization. This highlights the right hemisphere's role in adaptive responses to left-hemispheric gliomas and further underscores the importance of molecular profiling and tumor location in understanding reorganization potential.

## Teaser

Using machine learning, Strawderman *et al.* reveal that left-hemisphere gliomas systematically reshape the right-hemisphere functional connectome. Right-hemisphere functional connectivity patterns could reliably distinguish patients from controls, predict tumor presence in the left parietal cortex, and detect isocitrate dehydrogenase-mutations, a clinically-relevant tumor marker.

## Graphical Abstract

**Graphical Abstract:**
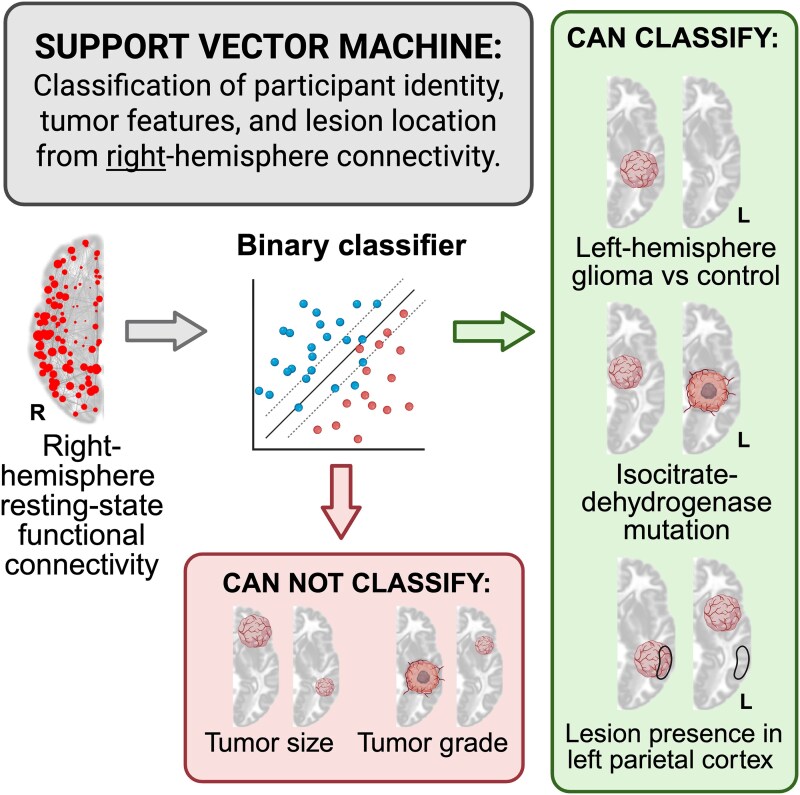

## Introduction

Functional reorganization in gliomas has important implications for understanding disease progression, neural plasticity, and patient outcomes.^1^ Gliomas infiltrate functional networks^2,3^ resulting in dynamic alterations in functional connectivity (FC),^4^ which may underlie both disruptions and preservation of cognition preoperatively in patients.^5,6^ Key factors that influence functional changes—such as tumor grade, genetic profile of the tumor (e.g. isocitrate dehydrogenase mutation), and anatomical location—in turn impact surgical planning, prognosis, and recovery of cognitive function after surgery.^6^ One way to examine glioma-driven functional changes is through the non-lesioned hemisphere, where BOLD signal alterations are not confounded by local effects of the tumor (e.g. neurovascular uncoupling, edema). Despite significant advances in mapping eloquent cognitive function before and during neurosurgery, relatively little progress has been made in understanding how the contralesional hemisphere may undergo functional reorganization in the presence of a unilateral tumor. To address this, we explored the systematic impact of gliomas on the contralesional resting-state functional connectome using fMRI collected in 48 pre-operative participants with left-hemisphere gliomas.

Glioma subtypes differ in their timelines of injury progression, with diffuse low-grade gliomas (LGGs) exhibiting slower proliferation (i.e. tumor momentum) and potentially allowing more time for functional reorganization to occur.^7^ Another factor that influences the pattern of reorganization is whether the glioma infiltrates or displaces healthy tissue. Tumor molecular markers are also increasingly recognized to impact FC and cognitive outcomes in glioma. For example, isocitrate dehydrogenase (*IDH*)-mutant gliomas are associated with greater contralesional grey matter volume,^8^ and fewer reductions in whole brain FC when compared with *IDH*-wildtype tumors.^9^ Both patterns suggest that *IDH*-mutation status modulates reorganization. *IDH*-mutant gliomas have also shown a significantly lower incidence of cognitive impairment when compared with *IDH*-wildtype gliomas^9-11^; since *IDH*-mutant tumors have less tumor momentum, this disparity in cognitive outcomes is potentially mediated by differences in functional reorganization as a compensatory mechanism.

Functional reorganization can be influenced by the anatomic location of the glioma.^12-18^ For instance, LGG infiltration of insular cortex has been associated with a significant increase of the contralesional insular resting-state FC with both hemispheres, especially visual and sensorimotor networks.^12^ Studies have also demonstrated increases in contralesional insular grey matter volume in patients with insular LGG.^13^ Similar findings have been observed with frontal gliomas.^15^ Other findings indicate the parietal lobe may play an important role in functional compensation.^4^ There is also evidence to suggest that tumors located in the left hemisphere are associated with more widespread impacts on FC when compared with right-sided gliomas.^18^ The current study builds on these findings by investigating right-hemisphere resting-state FC in patients with left-hemispheric gliomas.

Four patterns of functional reorganization are generally observed in glioma patients,^1^ including: (i) persistence of function within the tumor, (ii) reorganization within perilesional areas, (iii) ipsilesional reorganization, and (iv) contralesional reorganization. Regions homotopic (i.e. mirror-image) to the glioma have been argued to be critical hubs involved in patterns of broader reorganization within the contralesional hemisphere.^4,12-14,19^ Understanding the role of the contralesional hemisphere in the cascade of neural changes that are caused by gliomas has important implications for advancing knowledge of how the brain adapts to injury and for potential therapeutic directions (e.g. guided-plasticity).^20,21^ Thus, the primary aim of this study was to investigate which characteristics of gliomas in the left hemisphere are associated with changes in patterns of contralesional resting-state FC (i.e. distinct from controls). We first test this using a classifier to assess whether patterns of right-hemisphere FC successfully predict whether a glioma is present in the left hemisphere. We used three validation metrics to evaluate the performance of the classifier: accuracy, precision, and recall. Each metric provides a unique perspective on classifier performance. Accuracy measures the total percent of correct classifications, considering both true positives and true negatives. Precision indicates the likelihood that a positive prediction is correct, while recall assesses the model's ability to identify all positive cases. Relying solely on accuracy can be misleading when classes are imbalanced. Consistently high accuracy, precision, and recall beyond chance levels provides strong evidence of systematic differences in distal FC in left-hemisphere glioma patients.

We also examined glioma characteristics, including tumor location and biologic factors like tumor grade and *IDH*-mutation, which influence tumor momentum and infiltration patterns. We expect these factors to affect systematic changes in right-hemisphere FC, revealing insights into the interplay between glioma biology and contralesional reorganization.

## Materials and methods

### Participants

Fifty-three participants with a unilateral left-hemisphere glioma underwent pre-operative fMRI for the Program for Translational Brain Mapping at the University of Rochester. Participants with gliomas involving the corpus callosum (*n* = 3) or with significant midline shift (*n* = 2) were excluded, resulting in a total of 48 participants (mean age = 50.04; standard deviation = 16.74; 31 males). Our sample included 32 high-grade and 16 low-grade gliomas using the 2021 World Health Organization (WHO) grade classification. Genetic sequencing following neurosurgery revealed that 25 of the gliomas were *IDH*-mutant, and 23 of the gliomas were *IDH*-wildtype (see Table 1). All participants provided written informed consent in accordance with the University of Rochester Research Subjects Review Board and were compensated monetarily for their time volunteering.

**Table 1:** Demographic information of each participant with a left hemisphere glioma

Subject	Sex	Diagnosis^a^	*IDH* Mutation	WHO Grade^b^	Age	Lesion Volume^c^	# fMRI Runs
1	Male	Glioblastoma	Wildtype	High	73	11349	1
2	Male	Glioblastoma	Wildtype	High	69	31 102	1
3	Female	Glioblastoma	Mutant	High	58	188 060	1
4	Male	Glioblastoma	Wildtype	High	57	145 300	2
5	Male	Oligodendroglioma	Mutant	Low	46	67 034	2
6	Female	Glioblastoma	Mutant	High	45	277 109	2
7	Male	Anaplastic astrocytoma	Wildtype	High	42	44 270	2
8	Male	Oligodendroglioma	Mutant	High	67	42 216	2
9	Male	Glioblastoma	Wildtype	High	67	18 604	2
10	Male	Glioblastoma	Mutant	High	28	193 209	1
11	Female	Anaplastic astrocytoma	Mutant	High	36	64 557	2
12	Female	Glioblastoma	Wildtype	High	49	11 801	1
13	Female	Gemistocytic astrocytoma	Mutant	Low	55	15 297	2
14	Male	Diffuse astrocytoma	Mutant	Low	20	7570	2
15	Male	Diffuse astrocytoma	Mutant	Low	23	19 704	2
16	Male	Oligodendroglioma	Mutant	Low	57	9308	2
17	Male	Oligodendroglioma	Mutant	Low	32	19 057	2
18	Female	Anaplastic astrocytoma	Mutant	High	29	69 361	2
19	Female	Glioblastoma	Wildtype	High	75	60 841	2
20	Male	Glioblastoma	Wildtype	High	56	45 310	1
21	Male	Anaplastic astrocytoma	Mutant	High	28	62 731	1
22	Male	Anaplastic astrocytoma	Wildtype	High	70	102 302	1
23	Female	Glioblastoma	Wildtype	High	66	6054	1
24	Male	Glioblastoma	Wildtype	High	67	199 912	1
25	Female	Glioblastoma	Wildtype	High	52	28 292	1
26	Female	Oligodendroglioma	Mutant	Low	60	17 316	2
27	Male	Astrocytoma	Wildtype	Low	25	74 908	2
28	Male	Anaplastic oligodendroglioma	Mutant	High	31	66 304	2
29	Female	Angiocentric glioma	Wildtype	Low	24	8505	2
30	Male	Glioblastoma	Wildtype	High	59	116 479	2
31	Female	Oligodendroglioma	Mutant	Low	62	25 170	2
32	Male	Diffuse astrocytoma	Mutant	Low	45	110 326	2
33	Male	Astrocytoma	Wildtype	Low	70	19 704	2
34	Male	Glioblastoma	Wildtype	High	48	129 643	1
35	Female	Oligodendroglioma	Mutant	Low	40	12 465	1
36	Male	Glioblastoma	Wildtype	High	42	192 798	1
37	Male	Glioblastoma	Wildtype	High	76	1538	2
38	Male	Anaplastic oligodendroglioma	Mutant	High	28	37 022	2
39	Female	Glioblastoma	Wildtype	High	79	35 746	2
40	Male	Infiltrating astrocytoma	Mutant	Low	41	33 495	2
41	Male	Glioblastoma	Wildtype	High	63	16 919	2
42	Female	Oligodendroglioma	Mutant	Low	53	64 556	2
43	Female	Infiltrating glioma	Mutant	Low	43	23 096	1
44	Male	Anaplastic astrocytoma	Mutant	High	35	47 252	2
45	Female	Anaplastic astrocytoma	Mutant	High	40	26 814	2
46	Male	Glioblastoma	Wildtype	High	62	83 984	2
47	Male	Glioblastoma	Wildtype	High	75	96 475	2
48	Male	Anaplastic glioma	Mutant	High	34	71 566	2

^a^Pathology diagnosis obtained from biopsy report at time of surgery.

^b^As determined by 2021 World Health Organization (WHO) Grade Classification. High = Grade 3 and 4; Low = Grade 1 and 2.

^c^Number of voxels in standard space (MNI152NLin2009cAsym 1 mm^3^).

An additional 107 neurotypical adults (mean = 49.18; standard deviation = 15.87; 44 males) who did not differ in age from the glioma cohort (*z* = 0.27, *P* = 0.79, Wilcoxon rank-sum test with approximate *P*-value) underwent structural and resting-state fMRI. These subjects presented with complaints of subjective hearing loss, tinnitus, or headache and were referred by their physician to get imaging studies to rule out a structural cause. MRI examinations were evaluated by a neuro-radiologist (co-author S.P. Meyers) prior to all analyses herein and showed no signs of structural abnormality. This study was approved by the University of Rochester Research Subjects Review Board.

### MRI acquisition

Glioma participants were scanned on a 3-Tesla Siemens MAGNETOM Trio scanner with a 32 or 64-channel head coil (see Supplementary Table 1 for scanning parameters). Each session consisted of a T1-weighted (T1w) anatomical scan, and one to two runs of resting-state fMRI (Table 1). High-resolution T1w sagittal images were acquired using a magnetization-prepared rapid gradient echo pulse sequence at the start of scanning session. Whole brain BOLD imaging was acquired using a Gradient Echo Planar Imaging sequence.

Neurotypical participants were scanned on a 3-Tesla Siemens SKYRA scanner with a 20-channel head coil. Each session consisted of a T1w anatomical scan and 1 run of resting-state fMRI. High-resolution structural T1w and whole brain BOLD imaging were acquired as above. There were small variations in scanning parameters for this cohort due to the clinical nature of acquisition (Supplementary Table 1).

### Lesion identification

Lesions were segmented to distinguish healthy from abnormal tissue and reviewed by a neuro-radiologist (co-author S.P. Meyers) who was blinded to the study aims at the time. The neuro-radiologist had access to T1w (with and without contrast), T2w, and FLAIR imaging. Lesion renderings were inspected and corrected by the neuro-radiologist and study team to ensure accurate boundary delineation, with inclusion of edematous tissue. Lesion boundaries were defined in each subject's native T1w image, with abnormal voxels assigned a value of one and all others assigned a value of zero using ITK-SNAP. Participants provided written consent for the research team to access clinical MRI data.

**Figure 1:**
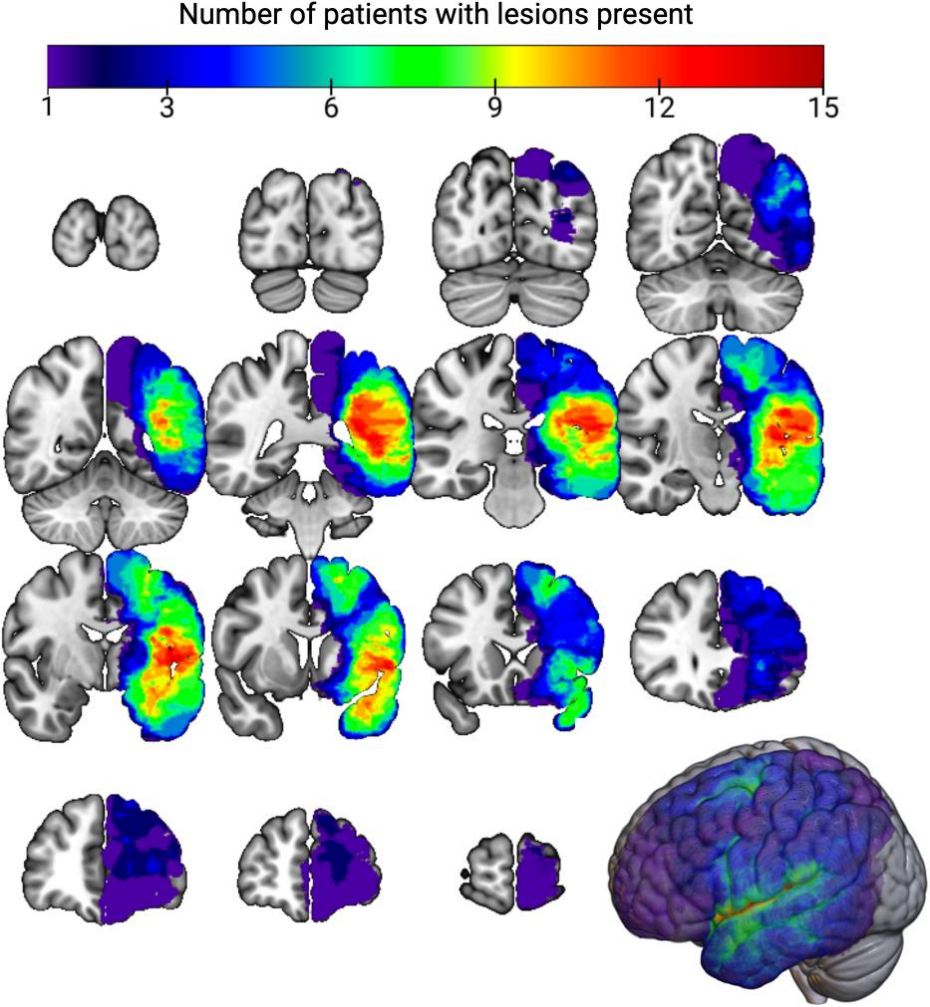
**Lesion overlap map.** Lesion overlap among the glioma cohort (*n* = 48 patients). We have broad coverage of the left hemisphere except for the occipital lobe. The maximal point of patient lesion overlap is *n* = 15 and centered around the left insula and operculum. Our cohort also has significant coverage of the anterior temporal lobe, inferior temporal gyrus, and superior frontal gyrus. Created in BioRender. Strawderman, E. (2025) https://BioRender.com/8bdnobs.

Lesion renderings in native T1w space were entered as cost function masks^22^ during anatomical and functional data pre-processing to ensure accurate normalization of anatomical data to Montreal Neurological Institute (MNI) space while minimizing lesion-related error. The corrected lesion drawings were then normalized to MNI space using the transformation matrix derived from the native T1w normalization (using the ANTs toolbox). Finally, the accuracy of the lesions in MNI space was reviewed and confirmed, referencing anatomical landmarks to ensure no distortions were introduced during normalization.^23-27^ Fig. 1 shows a lesion overlap map across all patients. The lesion distribution aligns with prior studies, with most lesions in frontal, temporal, parietal, and insular lobes.^28-30^

### MRI preprocessing

All MRI data were converted into Brain Imaging Data Structure format.^31^ The preprocessed T1w images were defaced.^32^ Anatomical and functional preprocessing steps were implemented using the standard protocol in fMRIPrep 21.0.1,^33^ based on NiPype 1.6.1^34^ (for details, see Supplementary material).

### Resting-state FC

FC is represented as a Fisher-transformed bivariate correlation coefficient from a weighted general linear model,^35^ defined separately for each ROI-to-ROI pair (ROI = region-of-interest). For every participant (patient and controls), we computed ROI-to-ROI FC among all right-hemisphere ROIs in the Yan homotopic atlas.^36^ The resulting right-hemisphere connectivity matrix was mean-normalized at the subject-level, resulting in a ROI-to-ROI connectivity matrix of *z*-scores (RRCz). This facilitates comparison across participants and focuses analyses on the variance in FC across the sample.^37^ Additionally, it accounts for magnitude differences in BOLD values across patients and controls that might result from inter-scanner differences—analyses are based on the patterns of variance in connectivity among ROIs, and how those patterns relates to characteristics of patients’ tumors.

### Classification of subjects as patients or controls based on right-hemisphere ROI-to-ROI connectivity

**Figure 2:**
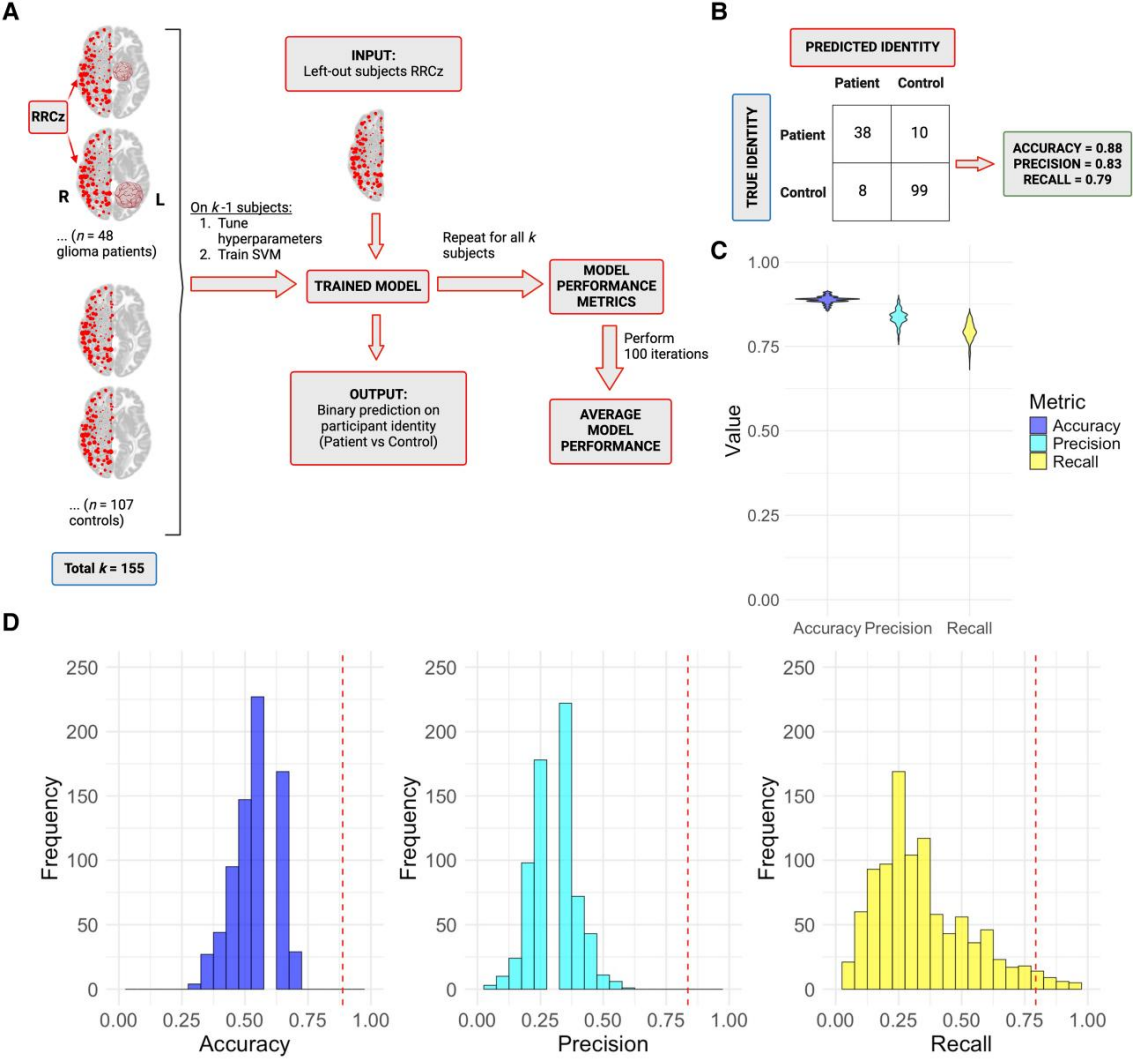
**The presence of a glioma in the left hemisphere is predicted by patterns of right-hemispheric FC.** (**A**) Schematic for participant identity support vector machine (SVM) pipeline. For a single iteration, we employed a linear SVM with nested cross-validation (external LOOCV to generate participant identity predictions for each left-out subject and internal 5-fold cross-validation for hyperparameter tuning; see Supplementary Fig. 1). Accuracy, precision, and recall were calculated from the confusion matrix comparing actual and predicted identity using formulas in Table 2 (see panel **B** for example). This process was repeated 100 times, generating a distribution of model performance for accuracy, precision, and recall. (**B**) Exemplar iteration's confusion matrix with accuracy, precision, and recall values closely matching average performance across all trials. Patients are considered the ‘positive’ class (i.e. a true positive is the top-left cell in the confusion matrix). (**C**) Violin plots of model performance across 100 iterations (accuracy: *x̄* = 0.89, *s* = 0.01; precision: *x̄* = 0.84, *s* = 0.02; recall: *x̄* = 0.79, *s* = 0.03). (**D**) Distributions of accuracy (*x̄* = 0.55, *s* = 0.08), precision (*x̄* = 0.31, *s* = 0.08), and recall (*x̄* = 0.35, *s* = 0.19) generated by permutation tests. The vertical lines indicate the mean values from Fig. 2C. Accuracy and precision have empirical *P* values of 0.001, whereas recall's *P* value is 0.028. Empirical *P*-values are determined from comparison of the mean model performance metric and the associated Monte-Carlo permutation distribution using the formula $P=\frac{r+1}{n+1}$, where *r* is the number of iterations ≥ mean model performance value and *n* = number of total iterations (*n* = 1000). For (**C**) and (**D**), distributions are shown on scale of possible model performance metrics (0 to 1) to facilitate assessment of model stability. For (**A**)—(**D**), the sample consisted of 155 participants (*n*_patients_ = 48, *n*_controls_ = 107). Created in BioRender. Strawderman, E. (2025) https://BioRender.com/qv1pn1p.

A support vector machine (SVM)^38^ was employed in MATLAB R2022a to classify participants as patients or controls based on ROI-to-ROI connectivity among the 100 right-hemisphere ROIs (feature space = 4950 edges). A nested cross-validation approach was implemented with leave-one-out cross-validation (LOOCV) in the outer loop for model evaluation, and 5-fold cross-validation in the inner loop for tuning the regularization parameter (Supplementary Fig. 1). Each iteration trained a linear SVM with L2-regularized dual stochastic gradient descent, which is optimized for high-dimensional data. Class imbalance was addressed with dynamic class weights recalculated for each external fold. Completion of the outer loop results in a vector of 155 binary predictions for participant identity. We generated a confusion matrix from the predicted and actual identity vectors and calculated accuracy, precision, and recall^39^ (Table 2). This process was repeated 100 times to calculate average model performance and assess model stability (Fig. 2A, see Supplementary material for full details). No feature selection was applied, as retaining all edges yielded optimal and robust performance.

**Table 2:** Performance metrics for binary classification models

Measure	Formula^a^	Interpretation
Accuracy	$\frac{TP+TN}{All}$	Proportion of all cases classified correctly.
Precision	$\frac{TP}{TP+FP}$	Proportion of the positively predicted cases that are truly positive (PPV^b^).
Recall	$\frac{TP}{TP+FN}$	Proportion of the truly positive cases that were correctly predicted (sensitivity).

^a^TP = true positive; TN = true negative; FP = false positive; FN = false negative.

^b^PPV = positive predictive value.

We used Monte Carlo permutation tests to estimate the significance of classification performance. Actual identity labels were randomly scrambled before applying the full classification procedure as described above. Accuracy, precision, and recall were calculated by comparing the prediction vector to the permuted identity vector. This was repeated for 1000 iterations to create sampling distributions for each metric. One-tailed empirical *P*-values for the average accuracy, precision, and recall values were calculated against their respective distributions ($P=\frac{r+1}{n+1}$, where *r* is the number of iterations ≥ actual value and *n* = number of iterations).^40^

### Mapping lesion locations of right-hemisphere connectivity-based patient clusters

**Figure 3:**
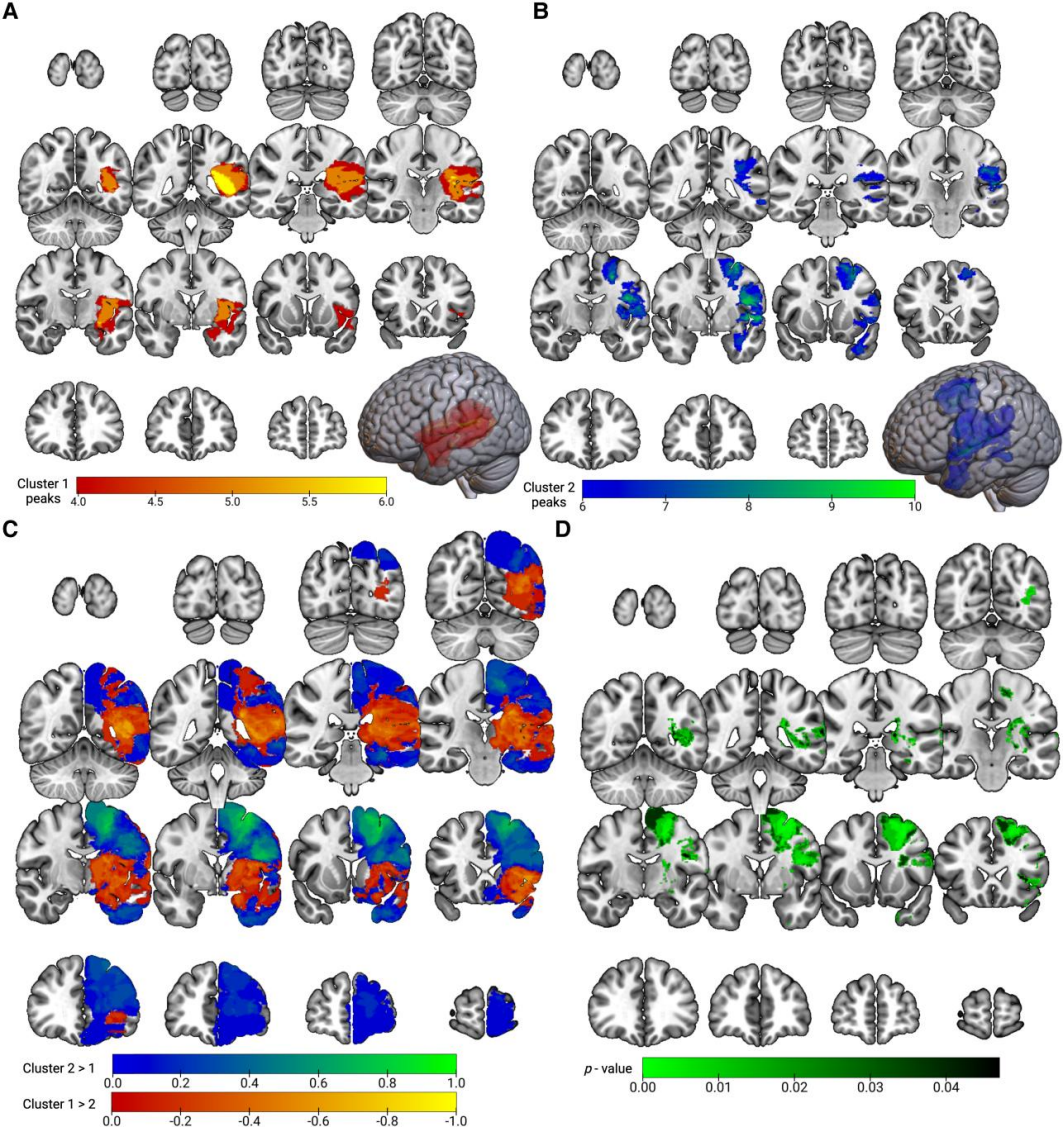
**Left-hemisphere lesion location associated with separable patterns of contralesional FC.** (**A**) Lesion overlap for patients in cluster one (*n* = 9 patients) thresholded to display the top half of values (number of overlapping patients range from 4 to 6). The point of maximal overlap is located in posterior perisylvian regions (e.g. planum temporale, parietal operculum) in addition to the white matter medial to these structures (i.e. arcuate fasciculus). (**B**) Lesion overlap for patients in cluster two (*n* = 39 patients) thresholded to display the top half of values (number of overlapping patients range from 6 to 10). There are three main peaks—posterior superior frontal gyrus, pars opercularis together with the frontal aslant tract, and anterior perisylvian regions including the central operculum and planum polare (**C**) Difference map between cluster two and cluster one (cluster two—cluster one). Prior to subtraction of the images in FSL, each un-thresholded image was divided by the maximal overlap (*n* = 6 and 10 for cluster one and two, respectively). This allowed for normalization on a scale from 0 to 1, accounting for differences in the number of subjects in each cluster. Positive values represent relative cluster two dominance (blue-green) and negative values represent relative cluster one dominance (red-yellow). (**D**) Uncorrected *P*-values from voxelwise Barnard's exact tests comparing lesion overlap between clusters, adjusting for unequal cluster sizes as in Fig. 3C. The *P*-values are thresholded at alpha = 0.05. Created in BioRender. Strawderman, E. (2025) https://BioRender.com/s7a7cge.

To identify subjects with similar right-hemispheric connectivity patterns, we performed *k*-means clustering on the patient ROI-to-ROI connectivity matrix (RRCz). The optimal number of clusters (*k*) was determined using silhouette analysis,^41^ which balances cluster cohesion (i.e. intra-cluster variance) and cluster separation (i.e. inter-cluster variance) to determine the optimal *k* (Supplementary Fig. 2). We then applied a *k*-means clustering algorithm to the full patient RRCz matrix. Each patient was then assigned to one of *k* clusters, and we subsequently constructed lesion overlap maps for each cluster (Fig. 3).

### Predicting regional lesion presence using right-hemisphere patterns of ROI-to-ROI connectivity

**Figure 4:**
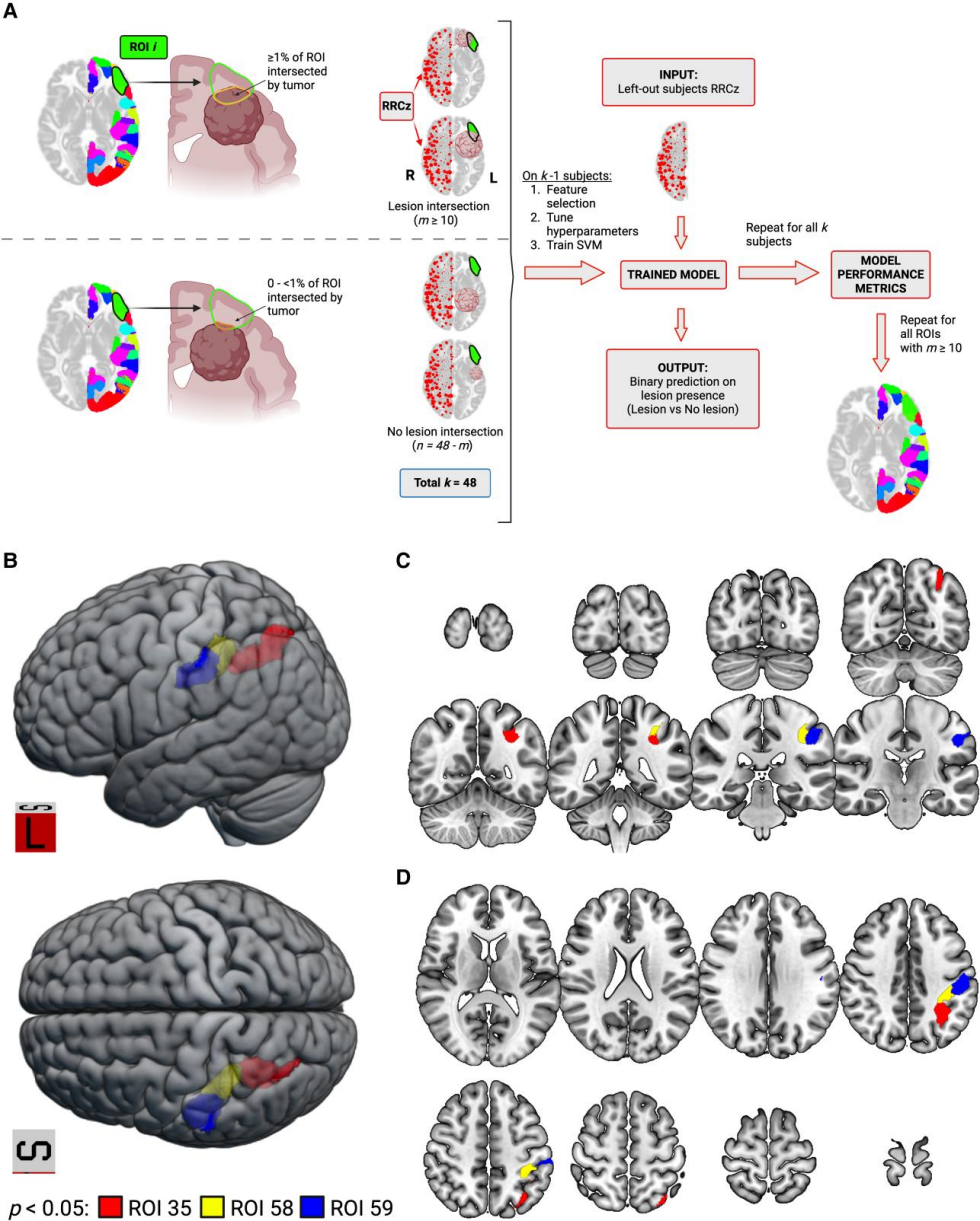
**Left-hemisphere gliomas involving parietal cortex are associated with systematic changes to right-hemisphere FC patterns.** (**A**) Schematic for ROI-specific lesion presence SVM pipeline. Lesion presence was binarized at a 1% threshold (i.e. a lesion was marked as present if it intersected ≥ 1% of the ROI's total volume). For each ROI with at least 10 patients with lesions present, we employed a linear SVM with nested cross-validation (external LOOCV to generate lesion presence predictions for each left-out subject and internal LOOCV for hyperparameter tuning; see Supplementary Fig. 1). Minimum Redundancy Maximum Relevance using an empirically derived feature importance threshold (defined separately for each ROI) was applied to select features independently for each training set of the external fold. Accuracy, precision, and recall were calculated from the confusion matrix comparing actual and predicted lesion presence using formulas in Table 2. This process was repeated for all suprathreshold ROIs. (**B**) There were three left-hemisphere ROIs for which tumor involvement was predicted by right-hemisphere FC. ROI_35_: accuracy = 0.77 (*P* = 0.048), precision = 0.55 (*P* = 0.047), recall = 0.5 (*P* = 0.038), MCC = 0.37 (*P* = 0.02). ROI_58_: accuracy = 0.79 (*P* = 0.013), precision = 0.63 (*P* = 0.019), recall = 0.42 (*P* = 0.049), MCC = 0.39 (*P* = 0.017). ROI_59_: accuracy = 0.81 (*P* = 0.006), precision = 0.69 (*P* = 0.009), recall = 0.64 (*P* = 0.006), MCC = 0.54 (*P* = 0.003). These regions are centered on the intraparietal sulcus and extend anteriorly into the postcentral gyrus. Shown in left-sagittal (top) and superior (bottom) renderings. MCC = Matthews correlation coefficient. (**C**) Coronal view of the significant ROIs. (**D**) Axial view of the significant ROIs. Created in BioRender. Strawderman, E. (2025) https://BioRender.com/th3371q.

We used the Yan atlas to define 100 anatomical parcels in each hemisphere.^36^ The atlas was resliced from FSLMNI152 1 mm^3^ space to MNI152NLin2009cAsym space using the nii_reslice_target function.^42^ For each left-hemisphere ROI, we calculated the proportion of lesion intersection per participant using in-house MATLAB scripts (Lesion ∩ ROI/ROI volume). Lesion intersection was binarized: ≥1% of ROI intersection was labelled as lesion-present (Fig. 4A). ROIs were included for subsequent analysis if 20% of patients (i.e. *n* ≥ 10) had lesions present to ensure a reasonable minimum class imbalance (1:4).

To assess whether lesion presence in an individual ROI could be predicted by right-hemisphere FC, we applied an SVM framework like the participant identity classification, with four key modifications. First, we incorporated feature selection using Minimum Redundancy Maximal Relevance (MRMR),^43^ which identifies features that are most correlated with the outcome and least correlated with each other. We hypothesized that fewer, spatially specific features would be sufficient to classify lesion presence in individual ROIs (e.g. due to homotopic redistribution).^4,12,44^ Second, due to the large number of ROIs, we used LOOCV for internal folds to ensure model stability rather than performing 100 iterations of the pipeline and calculating average performance. Third, to meaningfully compare classifier performance across ROIs—each with varying degrees of class imbalance—we calculated the Matthews correlation coefficient (MCC) as a unified metric.^45^ MCC is widely regarded as a robust, overall assessment of binary classifier performance, particularly for imbalanced datasets.^46,47^ Unlike other metrics, it accounts the entire confusion matrix, is invariant to the labelling of the positive class, and generates a high score only if the classifier can correctly predict the majority of positive cases and the majority of negative cases.^46,48^ Participant identity classification showed strong alignment across accuracy, precision, and recall, making MCC less relevant. In contrast, ROI-level lesion classification revealed metric discordance (e.g. high recall but low accuracy), emphasizing the importance computing MCC for each ROI-specific SVM. Finally, we used a two-stage Monte Carlo permutation testing approach: an initial 100-iteration screen to identify candidate ROIs based on significant MCC values, then a 1000-iteration permutation test for all candidate ROIs (*p*_MCC_ < 0.05 on the 100-iteration screen; see Supplementary material for details).

### Predicting tumor characteristics based on right-hemisphere patterns of ROI-to-ROI connectivity

To assess whether tumor characteristics could be predicted by right-hemisphere FC, we used the same SVM framework as the participant identity classification (i.e. external LOOCV and internal 5-fold CV), with the addition of MRMR feature selection to improve model stability given the smaller sample size (*n* = 48). This pipeline was applied to classify patients by (i) *IDH*-mutation (mutant versus wildtype), (ii) WHO Grade (high-grade versus low-grade), and as a control analysis, (iii) tumor volume (defined as small or large based on a median-split). See Supplementary material for details and Supplementary Table 2 for summary of all SVM approaches.

### Statistical analysis

Upon visual inspection of histograms, several variables did not meet assumptions of normality; therefore, non-parametric methods were applied where appropriate. Associations between continuous variables were assessed using two-tailed Spearman rank correlations (*ρ*_df_). Group comparisons were made using the two-tailed Wilcoxon rank-sum test; we reported the standardized test statistic (*z*) and normal-approximated *P*-value (given that min(*n_x_*, *n_y_*) ≥ 10).^49^ Significance of model performance was estimated using Monte Carlo permutation testing and empirical *P*-values (calculated by the more conservative formula, $P=\frac{r+1}{n+1}$).^40^ Other statistical methods (e.g. SVMs) are described separately in the Materials and methods above. Blinding and randomization were not feasible due to the retrospective, observational nature of the study. For all analyses, an alpha of 0.05 was utilized (*P* < 0.05).

## Results

### Left-hemisphere glioma presence is predicted by pattern of right-hemisphere FC

We constructed a binary classification model with nested cross-validation that predicted participant identity (glioma patient versus control) based on patterns of right-hemisphere ROI-to-ROI connectivity (RRCz; Fig. 2A). Model performance was evaluated by comparing predicted versus true identities, from which accuracy, precision, and recall were calculated (Fig. 2B). This procedure was repeated 100 times and average performance was calculated across trials (Fig. 2C). On average, the model achieved 89% accuracy, correctly identifying participants as patients or controls. Of the participants predicted to be patients, 84% were truly patients (precision), while 79% of patients were correctly identified as patients (recall; 21% misclassified as controls on average). For an example trial whose performance closely matched that of the average performance (see the confusion matrix in Fig. 2B), we found no association between lesion size, age, *IDH*-mutation status, and WHO grade with the likelihood of being misclassified (see Supplemental Material and Supplementary Table 3 for detail).

To confirm that the average performance of the model exceeded chance, we conducted permutation tests for each metric (Fig. 2D). Each of the mean performance metrics was highly significant (empirical *P* = 0.001 for accuracy and precision, *P* = 0.028 for recall), indicating that the model's ability to differentiate patients from controls is substantially greater than chance. Overall, these findings indicate that the presence of a left-hemisphere glioma is associated with consistent, classifiable differences in patterns of right-hemisphere connectivity.

### Left-hemisphere lesion location associated with separable patterns of contralesional FC

We hypothesized that left-hemisphere gliomas would differentially affect subsets of the 4950 right-hemisphere connectivity edges based on lesion location. To test this, we performed *k*-means clustering on patients based on right-hemisphere RRCz (*k* = 2, determined by a maximal silhouette coefficient, see Supplementary Fig. 2). Lesion overlap maps were generated for the patients in each cluster (Supplementary Fig. 3A, B). Cluster one (*n* = 9) exhibited a bullseye lesion distribution centered on the posterior perisylvian region, and progressively diminishing overlap in more distal regions (Supplementary Fig. 3A). Cluster two (*n* = 39) displayed broader coverage, with peaks in frontal and perisylvian regions (Supplementary Fig. 3B).

To delineate spatial differences in the patient clusters, we increased the signal-to-noise contrast by filtering each cluster's lesion overlap map such that only the top half of values in each cluster were visible (Fig. 3A, B). This approach demonstrates the tight spatial focus of cluster one around the Sylvian fissure, with a posterior predominance. The highest point of lesion overlap is in the white matter medial to the planum temporale and parietal operculum, in particular the temporal component of the superior longitudinal fasciculus/arcuate fasciculus (Fig. 3A). While some components of cluster two are also perisylvian, they are located anteriorly (e.g. central operculum, planum polare). Additionally, there are cluster two peaks located in posterior superior frontal gyrus, pars opercularis, and the frontal aslant tract (Fig. 3B).

To further highlight spatial differences between the clusters, we constructed cluster lesion overlap ‘difference maps’ such that positive values represent cluster two dominance (blue-green; Fig. 3C) and negative values represent cluster one dominance (red-yellow; Fig. 3C). To control for differences in sample size between clusters, we normalized each cluster map by its maximum overlap value before computing the difference (i.e. cluster two/max overlap_cluster two_ – cluster one/max overlap_cluster one_). This analysis highlights the perisylvian dominance of cluster one relative to cluster two once differences in relative magnitude are controlled. We then conducted voxel-wise Barnard's exact tests comparing lesion overlap between clusters, adjusting for unequal cluster sizes (for details, see Supplementary material). We first generated an uncorrected *P*-value map thresholded at alpha as an exploratory analysis (Fig. 3D). The regions with significant *P*-values align with the cluster peaks identified in Fig. 3A–C. These findings suggest that lesion presence within circumscribed regions may be associated with stereotyped patterns in contralesional FC; however, an important limitation is that no voxels survived multiple comparisons, underscoring the need for larger studies with sufficient power to confirm the relationship between lesion location and contralesional FC.

### Glioma presence in left parietal cortex is predicted by right-sided FC patterns

Given that patients with lesions in similar locations exhibited consistent contralesional connectivity patterns (Fig. 3), we hypothesized that distinct changes in contralesional FC may predict glioma presence in specific left-hemisphere regions. Using an SVM, we tested whether right-hemisphere RRCz could predict the presence of gliomas in individual left-hemisphere ROIs (Fig. 4A). This analysis was restricted to 42 suprathreshold left-hemisphere ROIs where at least 20% of patients had tumor involvement.

Three ROIs showed statistically significant accuracy, precision, recall, and MCC when compared with the permutation test distributions, indicating that right-hemisphere connectivity can reliably predict whether gliomas intersect those regions (Table 3). Significant ROIs form a contiguous cluster along the intraparietal sulcus, extending anteriorly into the postcentral gyrus (Fig. 4B–D). Within our sample, gliomas in these regions were linked to systematic, classifiable differences in contralesional FC.

**Table 3:** Right-hemisphere FC can predict glioma presence in the left parietal lobule

ROI	Anatomical region^a^	Accuracy (*P^b^*)	Precision (*P*)	Recall (*P*)	Matthews correlation coefficient^c^ (*P*)
35	Intraparietal sulcus	0.77 (0.048)	0.55 (0.047)	0.5 (0.038)	0.37 (0.02)
58	Postcentral gyrus	0.79 (0.013)	0.63 (0.019)	0.42 (0.049)	0.39 (0.017)
59	Postcentral gyrus	0.81 (0.006)	0.69 (0.009)	0.64 (0.006)	0.54 (0.003)

^a^As defined by the Yan homotopic atlas.

^b^Empirical *P*-values ($p=\;\frac{r+1}{n+1}$, where *r* is the number of iterations ≥ actual value and *n* = number of iterations) determined by comparison of observed model performance metrics to distribution generated by permutation tests (1000 iterations).

^c^MCC = $\frac{TP\;\times \;TN\;-\;FP\;\times \;FN}{\sqrt{\left(TP+FP)(TP+FN)(TN+FP)(TN+FN\right)}}$ TP = true positives, TN = true negatives, FP = false positives, FN = false negatives.

### Homotopic connectivity modulates the effect of glioma on contralesional FC

A key question raised by our findings is why only certain left-hemisphere tumor locations are associated with reliable changes in right hemisphere connectivity. A control analysis established that across ROIs, MCC is not significantly associated with the number of subjects with lesions present in individual left-hemisphere ROI (Supplementary Fig. 4). In other words, the observed differences in model performance between ROIs is not simply an artifact of the degree of class imbalance, suggesting that intrinsic properties of the significant parietal ROIs regions may underlie the effect.

Homologous regions can play a key role in functional reorganization in response to unilateral lesions.^4,8,12-14,19^ Thus, we hypothesized that regions more strongly connected to their homologs would show greater alterations in contralesional connectivity, as indexed by the ROI-specific model's ability to classify subjects by lesion presence. To obtain an unbiased measure of interhemispheric connectivity, we calculated the average homotopic connectivity in age-matched controls (*n* = 107) for each of the 42 homotopic ROI pairs. We found that model performance (MCC) was modestly associated with the average homotopic connectivity values across ROIs (Spearman *ρ*_40_ = 0.35; *P* = 0.022; Supplementary Fig. 5A). To assess the specificity of the effect, we also examined whether it extended to non-homotopic connectivity. Remarkably, average FC between each left hemisphere ROI and the 99 non-homotopic right hemisphere ROIs was not significantly associated with model performance across the 42 ROIs (Spearman *ρ*_40_ = 0.11; *P* = 0.49; Supplementary Fig. 5B). These findings suggest that regions more tightly coupled to the contralesional homolog specifically may be more likely to influence systematic changes in right-hemisphere connectivity when involved with left-hemisphere tumors.

### Contralesional FC can detect isocitrate dehydrogenase mutation status

Isocitrate dehydrogenase mutation is a key biological variable that stratifies glioma patients’ prognosis. *IDH*-mutant tumors are generally less aggressive than *IDH*-wildtype due to impaired metabolism, resulting in a slower rate of proliferation.^50^ Numerous studies have documented differences in the degree to which functional reorganization occurs as a function of *IDH*-mutation status, such that functional reorganization is more likely to occur in patients with *IDH*-mutant tumors.^10,11,51,52^ Given this, we hypothesized that contralesional FC should predict whether a patient is *IDH*-mutant or *IDH*-wildtype.

**Figure 5:**
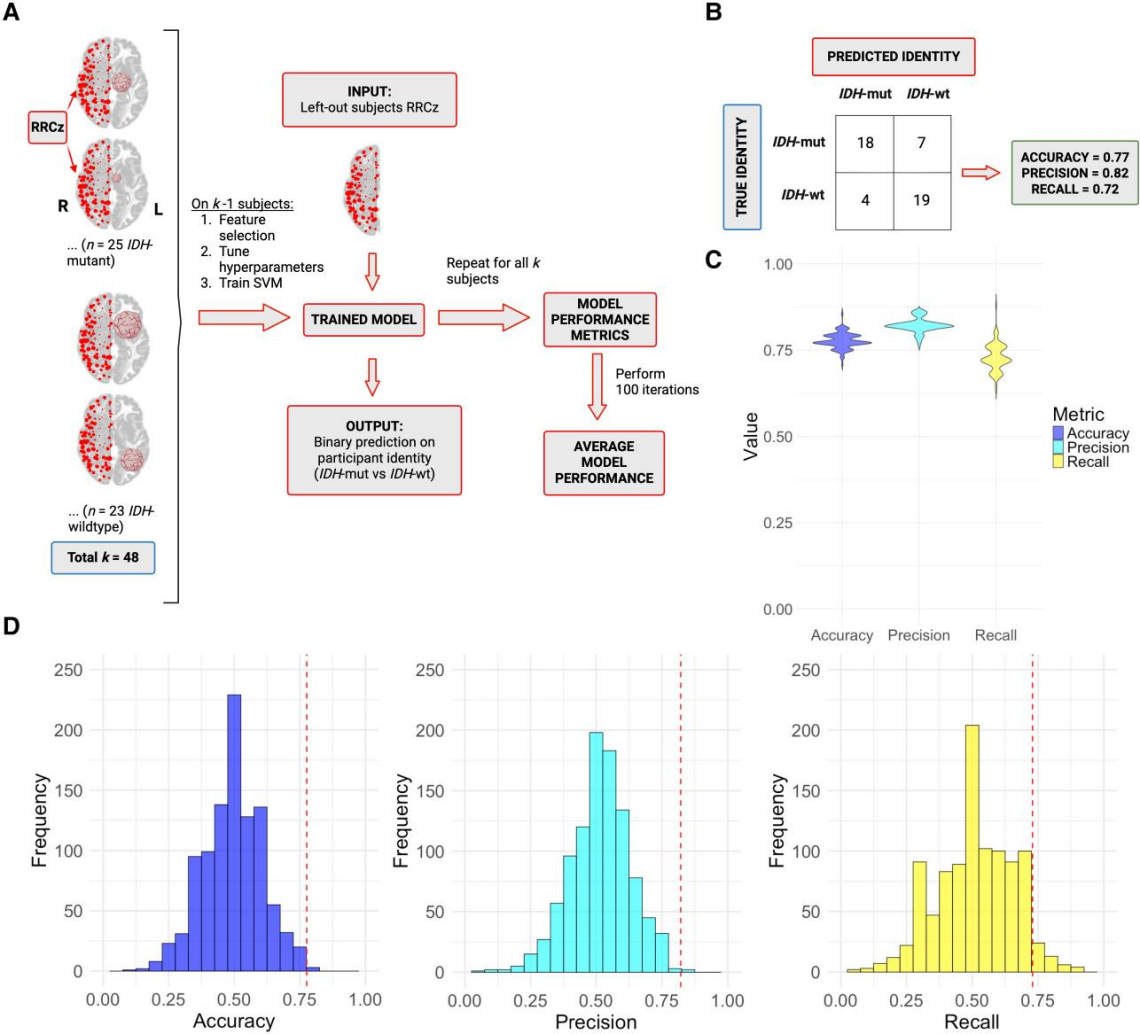
***IDH*-mutation status of left-hemisphere gliomas is predicted by patterns of right-hemisphere connectivity.** (**A**) Schematic for *IDH*-mutation support vector machine (SVM) pipeline. For a single iteration, we employed a linear SVM with nested cross-validation (external LOOCV to generate *IDH*-mutation predictions for each left-out subject and internal 5-fold cross-validation for hyperparameter tuning, see Supplementary Fig. 1). Minimum Redundancy Maximum Relevance using an empirically derived feature importance threshold of 0.05 was applied to select features independently for each training set of the external fold. Accuracy, precision, and recall were calculated from the confusion matrix comparing actual and predicted identity using formulas in Table 2 (see panel **B** for example). This process was repeated 100 times, generating a distribution of model performance for accuracy, precision, and recall (see panel C). **(B)** Exemplar iteration's confusion matrix with accuracy, precision, and recall values closely matching the average performance. *IDH*-mutant tumors are considered the ‘positive’ class (i.e. a true positive is the top-left cell in the confusion matrix). (**C**) Violin plots of model performance across 100 iterations (accuracy: *x̄* = 0.78, *s* = 0.02; precision: *x̄* = 0.82, *s* = 0.02; recall: *x̄* = 0.73, *s* = 0.04). (**D**) Distributions of accuracy (*x̄* = 0.50, *s* = 0.11), precision (*x̄* = 0.52, *s* = 0.12), and recall (*x̄* = 0.51, *s* = 0.14) generated by permutation tests. The vertical lines indicate the mean values from Fig. 5C. All model performance metrics are statistically significant against chance, with empirical *p*_accuracy_ = 0.004, *p*_precision_ = 0.003, and *p*_recall_ = 0.048. Empirical *P*-values are determined from comparison of the mean model performance metric and the associated Monte-Carlo permutation distribution using the formula $P=\frac{r+1}{n+1}$, where *r* is the number of iterations ≥ mean model performance value and *n* = number of total iterations (*n* = 1000). For (**C**) and (**D**), distributions are shown on scale of possible model performance metrics (0 to 1) to facilitate assessment of model stability. For (**A**)—(**D**), the sample consisted of 48 glioma patients (*n_IDH_*_–mut_ = 25, *n_IDH_*_-wt_ = 23). Created in BioRender. Strawderman, E. (2025) https://BioRender.com/5167uxn.

We trained SVMs to predict *IDH*-mutation status from the RRCz matrix (Fig. 5A, B). Across 100 iterations, we obtained average model performance metrics of 78% accuracy, 82% precision, and 73% recall (Fig. 5C). To assess whether these results are significantly better than chance, we performed permutation tests as described previously (Fig. 5D). All performance metrics reached statistical significance (*p*_accuracy_ = 0.004, *p*_precision_ = 0.003, and *p*_recall_ = 0.048). These findings align with prior evidence that the biologically distinct *IDH*-mutation cohorts differ in functional reorganization, supporting the hypothesis that these distal resting-state connectivity changes reflect reorganization.

A potential concern is that *IDH*-wildtype tumors, on average, tend to be larger than *IDH*-mutant tumors.^53^ This raises the possibility that the model is classifying patients by differences in tumor size and the extent to which left hemisphere regions are impacted, rather than *IDH*-mutation specifically. To examine this, we compared tumor volumes (calculated from MNI-space lesion files for consistency) between *IDH*-mutant and *IDH*-wildtype groups and found no significant difference (*z* = −0.01, *P* = 0.99, Wilcoxon rank-sum test with approximate *P*-value; Supplementary Fig. 6A).

Next, we tested whether relative tumor volume could be predicted from contralesional connectivity. Each patient's lesion was classified as ‘large’ or ‘small’ if it was greater or smaller, respectively, than the median lesion volume across patients. Average classification accuracy (36%), precision (35%), and recall (33%) were not significantly different from chance (all *P* ≥ 0.87; Supplementary Fig. 6B, C), suggesting that the ability to predict *IDH*-mutation status based on right-hemisphere connectivity is due to differences in tumor momentum or other intrinsic biological properties (e.g. synaptogenicity),^2^ rather than tumor size per se.

Like *IDH*-mutant tumors, low-grade gliomas are considered to have a greater likelihood of causing changes in FC.^7,54^ Therefore, we tested if the patterns of connectivity in the right hemisphere would be effective in classifying patients according to their WHO grade. We performed the same binary classification analysis using high-grade or low-grade as the outcome variable. This analysis showed that right-hemisphere RRCz could not effectively differentiate WHO grade across patients (*p*_accuracy_ = 0.63, *p*_precision_ = 0.58, *p*_recall_ = 0.55; Supplementary Fig. 7A–C). This finding, together with the null effects for size, suggests the success of the model in predicting *IDH*-mutation is due specifically to the effect of *IDH*-mutation driving functional changes.

## Discussion

Our findings indicate that left-hemisphere gliomas induce systematic alterations in right-hemisphere FC. We reasoned that if properties of gliomas are predictable from the contralesional connectome, it would represent a new way to understand how gliomas cause changes in the brain distal to the primary lesion. We leveraged machine learning techniques to demonstrate that patterns of right hemispheric ROI-to-ROI connectivity can accurately classify participants as patients or controls, predict lesion presence in the left parietal lobule, and differentiate patients based *IDH*-mutation status. These findings underscore the impact of gliomas on functional brain networks and provide novel insights into factors influencing lesion-induced functional reorganization.

Using a predictive approach allows for an unbiased determination of whether systematic differences exist in the connectome between glioma patients and controls at the population level. The 89% accuracy of our model in distinguishing patients from controls indicates that left hemisphere gliomas are associated with consistent, measurable changes in right-hemisphere connectivity (Fig. 2). While our data lack inherent causal directionality, three factors support the inference that the development of gliomas influence functional networks, not vice versa. First, prior studies align with the hypothesis that gliomas cause reorganization of adjacent and distal networks.^1,2,8,10,12-15,17,18,21,44,55,56^ Second, premorbid differences in FC are unlikely to ‘cause’ gliomas to form in the contralesional hemisphere. Finally, we focused on the non-lesional hemisphere only and used a robust control group to establish baseline connectivity, mitigating concerns of neurovascular uncoupling^57^ and increased hemodynamic lag^44^ within lesioned tissue. Given these considerations, it is reasonable to conclude that right-hemisphere patterns of connectivity in glioma patients would have mirrored controls pre-gliomagenesis, and the observed changes are the result of glioma growth in the left hemisphere.

Gliomas drive functional reorganization through both local plasticity near the tumor and global network adjustments. Those network changes are likely a result of molecular and cellular mechanisms. For instance, a recent study demonstrated the modulatory role of thrombospondin-1, a synaptogenic factor, on the functional remodeling of neural circuits induced by glioma.^2^ While the techniques utilized herein do not elucidate the underlying molecular mechanisms that contribute to functional reorganization, our findings are in line with the general hypothesis that functional changes in the contralesional hemisphere depend in part on factors such as tumor location and molecular markers.

Our cluster analysis findings emphasize that glioma location may differentially affect the contralesional connectome. By clustering patients by right hemisphere connectivity, we identified two distinct spatial patterns of left hemisphere lesions. One cluster showed tight spatial overlap in regions linked to language perception, such as the planum temporale and arcuate fasciculus (Fig. 3A). The second cluster localized to motor-relevant structures, including the superior frontal gyrus, pars opercularis, frontal aslant tract, and anterior insula (Fig. 3B). Notably, the frontal aslant tract connects the supplementary motor area in the superior frontal gyrus to the pars opercularis and the anterior insula,^58,59^ suggesting that this cluster involves multiple regions within a frontal language and action production *network*. That is, lesions separated in space but linked through a shared functional network can result in similar contralesional effects. Anatomically stereotyped changes in FC according to glioma location, as seen in cluster one, is consistent with prior theories of functional reorganization, such as reorganization to the homotopic region^4,12,44^; however, the multiple regions of lesion overlap within a functional network in cluster two expands upon this framework to suggest that the specific functions and network roles of affected left-hemisphere regions plays a substantial role in shaping contralesional connectivity.

We also found that the pattern of right hemisphere connectivity could predict the presence of a lesion in specific regions within the left hemisphere. Notably, tumors in the intraparietal sulcus and postcentral gyrus (Fig. 4) could be predicted by right hemisphere connectivity patterns. One hypothesis for what underlies that spatial specificity is intrinsic differences in interhemispheric connectivity. Interhemispheric connectivity varies significantly across the brain, such that regions with bilateral functions tend to exhibit greater interhemispheric connectivity compared with those with strongly lateralized functions.^60^ The regions found to be associated with classifiable contralesional changes are involved in some bilateral functions (e.g. the intraparietal sulcus for working memory^61,62^ and arithmetic/numerical processing,^63,64^ and postcentral gyrus for bilateral hand representation^65^). We thus tested whether interhemispheric connectivity is related to the degree to which lesion presence can be predicted by right hemisphere connectivity (Supplementary Fig. 5). To avoid bias introduced by tumor presence, we first calculated measures of homotopic connectivity in the control dataset to establish an approximate ‘ground truth’ of interhemispheric connectivity. We found that the Matthew's correlation coefficient (the gold standard measure of overall binary classifier performance)^46,47^ was modestly associated with the degree of homotopic connectivity across ROIs (Spearman *ρ*_40_ = 0.35; *P* = 0.022); however, model performance was not significantly associated with non-homotopic connectivity. These findings suggest that areas with higher homotopic connectivity may be more likely to recruit the contralateral hemisphere when lesioned. To our knowledge, this is the first study to report a potential link between the *strength* of homotopic connectivity and changes in the contralesional connectome, despite the role of the homologous region having been extensively studied in glioma patients.^4,12-14,19^ Additionally, Daniel and colleagues found that homotopic connectivity was associated with overall survival in glioma patients.^44^ Taken together, further work characterizing the relation between homotopic connectivity strength and contralesional recruitment in larger cohorts of patients may be of significant prognostic value. Additionally, the work herein focuses primarily on interhemispheric connectivity, but future work can include exploration of connectivity within the ipsilesional hemisphere, with particular emphasis on how lesions that affect ‘hubs', may affect network organization and dynamics.^66^

We further demonstrated that right-hemisphere connectivity can classify glioma patients based on *IDH*-mutation status with above-chance accuracy, precision, and recall, suggesting that *IDH*-mutant patients generally exhibit distinct contralesional connectivity patterns. This aligns with prior evidence that *IDH*-mutant tumors are associated with greater functional reorganization than *IDH*-wildtype tumors.^7-11,52^ This difference is thought to stem from variations in tumor growth dynamics rather than size alone, as the slower growth of *IDH*-mutant tumors may permit more extensive structural and functional adaptation.^7,11^ For instance, prior work found that neurocognitive function was inversely related to tumor size only within the *IDH*-wildtype cohort (whereas *IDH*-mutant patients had no relation between tumor size and lesion volume), underscoring the impact of rate of tumor growth on neurocognitive function.^11^ Notably, our finding that right-hemisphere connectivity could not predict tumor size further supports this interpretation, effectively ruling out tumor volume as the primary driver of the observed connectivity differences between *IDH*-mutant and *IDH*-wildtype patients.

Low-grade and high-grade gliomas also differ in tumor momentum, with high-grade gliomas having a faster rate of proliferation. Despite this, we found that the right hemisphere connectome could not accurately distinguish between low-grade and high-grade gliomas (Supplementary Fig. 7). In part, this may reflect an underpowered low-grade glioma cohort (*n* = 16). Alternatively, tumor biology specific to *IDH*-mutation may play a significant role in lesion-induced reorganization, independent of tumor grade. Recent work by van Kessel and colleagues^67^ found that tumor associated gene expression and effects on local metabolic processes are related to the cognitive effects of glial tumors; for example, expression of markers involved in synaptic function and plasticity (e.g. semaphorin-3A and neuroligin-3A) was associated with better neurocognitive performance, independently of the size, grade, and location of the tumors. Additionally, a metabolic byproduct of mutated *IDH* (D-2-hydroxyglutarate) is known to inhibit DNA demethylases, leading to altered gene transcription.^68^ It's possible that this altered pattern of gene expression could influence local neuroplasticity processes; however, the link between D-2-hydroxyglutarate and synaptic plasticity has not been explicitly studied to date and represents an interesting avenue for future work. Lastly, *IDH*-mutation can influence tumor location,^69-71^ shaping the extent and pattern of functional reorganization. We observed differences in the overall localization of lesion overlap based on *IDH*-mutation (Supplementary Fig. 8A). Interestingly, we found that the peak overlap for cluster one and *IDH*-wildtype tumors co-localize (Supplementary Fig. 8B), whereas the peak overlaps for cluster two and *IDH*-mutant tumors were highly similar (Supplementary Fig. 8C). Future research can explore whether *IDH*-mutation may shape functional reorganization through influencing anatomical location,^69^ in addition to biologic and momentum factors.^10,11,52^

Prior work on reorganization caused by glioma has focused on diffuse lower- and intermediate-grade gliomas.^4,5,7,12,14,54,72,73^ Our results emphasize the importance of considering molecular markers in addition to grade. This is in line with previous research showing significant structural and functional brain-wide changes in *IDH*-mutant gliomas.^8,11,74^ For example, Huang and colleagues found that contralesional macrostructural plasticity is observed in left insular *IDH*-mutant gliomas but had no association with histological grade, 1p19q co-deletion, or TERT promoter mutation.^8^ Notably, our relatively small sample size restricts our ability to draw conclusive insights about the differential impact of *IDH* mutation and WHO grade on the contralesional connectome. Our exploratory findings support the consensus to consider *IDH*-genotype alongside WHO grade when prognosticating neurocognitive and functional outcomes in patients,^75^ and motivates future work replicating these findings in a larger sample.

Our study has important limitations to consider. First, selection bias exists in our sample, as most patients in our brain mapping program have tumors near eloquent cortical regions. This selection bias may have influenced the clustering analysis and limits understanding of contralesional impacts outside our lesion coverage. Additionally, restricting the analysis to left hemisphere tumors (another byproduct of selection bias) prevents extrapolation to the right hemisphere, where functional network alterations may differ.^14^ Our expectation is that such lateralized effects of gliomas on contralesional connectivity may interact with whether impacted region supports functions represented unilaterally or bilaterally.

Across patients, we found no relationship between clinical variables (age, lesion volume, *IDH* mutation, and WHO grade) and misclassification as a control in the participant identity SVM (Supplementary Table 3). However, this analysis is limited by the number of misclassified patients (*n* = 10) to draw conclusions from.

A small subset of patients had signs of mild to moderate mass effect on their native T1w images that resolved with non-linear transformation to standard space. However, it's important to recognize that contralesional compression may still impact the functional connectome. One argument that mitigates this concern is patients with mass effect typically have larger tumors, yet the models could not classify tumors by size—suggesting mass effect did not systematically alter contralesional connectivity in this sample. Nonetheless, an important topic for future research is to understand how mechanical deformation of the brain may affect FC.

Another limitation is that patient and control MRI data were acquired on different scanners (see Methods, Supplementary Table 1). To address this, we used normalized ROI-to-ROI connectivity at the subject-level to minimize systematic differences in connectivity strength, which could relate to use of different MR scanners. Additional supporting analyses show that subject-level normalization of ROI-to-ROI connectivity eliminates differences between patients and controls in the overall distributions of connectivity values (see Supplementary Fig. 9 for analysis and discussion). Furthermore, the *IDH*-mutation model's performance cannot be explained by scanner-effects. Lastly, this study was cross-sectional and thus is based on an inference of the direction of causality (i.e. that gliomas cause changes in FC). Future work with longitudinal fMRI within the same patients offers a powerful approach to clarify the direction of causality from glioma growth to network reorganization. While reorganization is often presumed to preserve function normally performed by the lesioned tissue,^55^ future work should include cognitive data to confirm this association.

Despite these limitations, the results of our novel method to study functional reorganization are consistent with a growing body of evidence demonstrating that gliomas can induce changes in FC in distal regions, including the contralateral hemisphere^1,4,9,12,14,16,76^ and highlights the significance of isocitrate dehydrogenase mutation when considering the potential for functional reorganization in glioma patients. These conclusions hold considerable potential for prognostic importance. Future research incorporating molecular/genetic features, neuroimaging, and neurocognitive data in pre-operative glioma patients is needed to tease out the complex relation among molecular mechanisms, tumor momentum, interhemispheric connectivity, and lesion location to develop a cohesive framework underlying functional reorganization in glioma patients.

## Supplementary Material

fcaf349_Supplementary_Data

## Data Availability

All connectivity data and scripts used in preparation for this manuscript are available via Figshare: https://doi.org/10.1184/R1/29932124.
