# Supplementary material for: Left-hemisphere glioma drives systematic patterns of contralesional functional connectivity
Source: Brain Commun. 2025 Sep 11;7(5):fcaf349. doi: 10.1093/braincomms/fcaf349 (PMC12464944; doi:10.1093/braincomms/fcaf349)
Supplement: fcaf349_Supplementary_Data [file fcaf349_supplementary_data.pdf]

Left-hemisphere glioma drives systematic patterns of contralesional functional connectivity

Emma Strawderman [1,2], Frank E. Garcea[1,2], Madalina E. Tivarus[1,3], Steven P. Meyers[1-3], Adnan A. Hirad[1,4], William M. Burns[2], Kevin A. Walter[2], Tyler Schmidt[2], Webster H. Pilcher[2], and Bradford Z. Mahon[2,5]

1. Department of Neuroscience, University of Rochester Medical Center, Rochester, NY, 14624, USA
2. Department of Neurosurgery, University of Rochester Medical Center, Rochester, NY, 14624, USA
3. Department of Imaging Sciences, University of Rochester Medical Center, Rochester, NY, 14624, USA
4. Department of Surgery, University of Rochester Medical Center, Rochester, NY, 14624, USA
5. Department of Psychology, Carnegie Mellon University, Pittsburgh, PA, 15213, USA

Corresponding author:
Bradford Z. Mahon
Department of Psychology
Carnegie Mellon University
5000 Forbes Avenue
Pittsburgh, PA 15213, USA
bmahon@andrew.cmu.edu

Table of contents

Supplementary Methods (p. 2-13)
Supplementary Results (p. 13-14)
Supplementary Figures (p. 15-23)
Supplementary References (p. 24-25)

Supplementary Methods

Supplementary Table 1. Scanning Parameters

Subject			Anatomical Scan[a]				Functional Scan					
ID	P/C[b]	Head Coil	TR[c]	TE[d]	Flip Angle	Matrix	TR[c]	TE[d]	Flip Angle	Matrix	Voxel Size	Slices
1	P	64	2.5	2.3	7	256	2.2	30	70	128	2	90
2	P	64	2.5	2.3	7	256	2.2	30	70	128	2	90
3	P	32	2.5	3.4	7	256	2.2	30	90	64	4	33
4	P	32	2.5	3.4	7	256	2.2	30	90	64	4	33
5	P	64	2.5	2.3	7	256	2.2	30	70	128	2	90
6	P	64	2.3	2.3	8	256	2.2	30	70	128	2	90
7	P	32	2.5	3.4	7	256	2.2	30	90	64	4	33
8	P	64	2.5	2.3	7	256	2.2	30	70	128	2	90
9	P	32	2.5	3.4	7	256	2.2	30	90	64	4	33
10	P	32	2.5	3.4	7	256	2.2	30	90	64	4	33
11	P	32	2.5	3.4	7	256	2.2	30	90	64	4	33
12	P	32	2.5	3.4	7	256	2.2	30	90	64	4	33
13	P	32	2.5	3.4	7	256	2.2	30	90	64	4	33
14	P	64	2.5	2.3	7	256	2.2	30	70	128	2	90
15	P	64	2.5	2.3	7	256	2.2	30	70	128	2	90
16	P	64	2.5	2.3	7	256	2.2	30	70	128	2	90
17	P	64	2.5	2.3	7	256	2.2	30	70	128	2	90
18	P	64	2.5	2.3	7	256	2.2	30	70	128	2	90
19	P	64	2.5	2.3	7	256	2.2	30	70	128	2	90
20	P	64	2.5	2.3	7	256	2.2	30	70	128	2	90
21	P	64	2.5	2.3	7	256	2.2	30	70	128	2	90
22	P	64	2.5	2.3	7	256	2.2	30	70	128	2	90
23	P	64	2.5	2.3	7	256	2.2	30	70	128	2	90
24	P	64	2.5	2.3	7	256	2.2	30	70	128	2	90
25	P	64	2.5	2.3	7	256	2.2	30	70	128	2	90
26	P	32	2.5	3.4	7	256	2	30	90	64	4	30
27	P	32	2.5	3.4	7	256	2	30	90	64	4	30
28	P	64	2.5	2.3	7	256	2.2	30	70	128	2	90
29	P	32	2.5	3.4	7	256	2.2	30	90	64	4	33
30	P	32	2.5	3.4	7	256	2.2	30	90	64	4	33
31	P	32	2.5	3.4	7	256	2.2	30	90	64	4	33
32	P	32	2.5	3.4	7	256	2.2	30	90	64	4	33
33	P	32	2.5	3.4	7	256	2.2	30	90	64	4	33
34	P	64	2.5	2.3	7	256	2.2	30	70	128	2	90
35	P	64	2.5	2.3	7	256	2.2	30	70	128	2	90
36	P	32	2.5	3.4	7	256	2.2	30	90	64	4	33
37	P	32	2.5	3.4	7	256	2.2	30	90	64	4	33
38	P	32	2.5	3.4	7	256	2.2	30	90	64	4	33

39	P	32	2.5	3.4	7	256	2.2	30	90	64	4	33
40	P	64	2.5	2.3	7	256	2.2	30	70	128	2	90
41	P	64	2.5	2.3	7	256	2.2	30	70	128	2	90
42	P	64	2.5	2.3	7	256	2.2	30	70	128	2	90
43	P	64	2.5	2.3	7	256	2.2	30	70	128	2	90
44	P	64	2.5	2.3	7	256	2.2	30	70	128	2	90
45	P	64	2.5	2.3	7	256	2.2	30	70	128	2	90
46	P	64	2.5	2.3	7	256	2.2	30	70	128	2	90
47	P	64	2.5	2.3	7	256	2.2	30	70	128	2	90
48	P	32	2.5	3.4	7	256	2.2	30	90	64	4	33
49	C	20	1.2	2.3	8	256	2.4	30	90	74	3	42
50	C	20	1.2	2.3	8	256	2.9	30	90	74	3	50
51	C	20	1.2	2.3	8	256	2.4	30	90	74	3	42
52	C	20	1.2	2.3	8	256	2.4	30	90	74	3	42
53	C	20	1.2	2.3	8	256	2.4	30	90	74	3	42
54	C	20	1.2	2.3	8	256	2.4	30	90	74	3	42
55	C	20	1.2	2.3	8	256	2.4	30	90	74	3	42
56	C	20	1.2	2.3	8	256	2.4	30	90	74	3	42
57	C	20	1.2	2.3	8	256	2.4	30	90	74	3	42
58	C	20	1.2	2.3	8	224	2.4	30	90	74	3	42
59	C	20	1.2	2.3	8	256	2.4	30	90	74	3	42
60	C	20	1.2	2.3	8	256	2.4	30	90	74	3	42
61	C	20	1.2	2.3	8	256	2.4	30	90	74	3	42
62	C	20	1.2	2.3	8	256	2.4	30	90	74	3	42
63	C	20	1.2	2.3	8	256	2.4	30	90	74	3	42
64	C	20	1.2	2.3	8	256	2.4	30	90	74	3	42
65	C	20	1.2	2.3	8	256	2.4	30	90	74	3	42
66	C	20	1.2	2.3	8	256	2.4	30	90	74	3	42
67	C	20	1.2	2.3	8	256	2.4	30	90	74	3	42
68	C	20	1.2	2.3	8	256	2.4	30	90	74	3	42
69	C	20	1.2	2.3	8	256	2.4	30	90	74	3	42
70	C	20	1.2	2.3	8	256	2.8	30	90	74	3	48
71	C	20	1.2	2.3	8	256	2.4	30	90	74	3	42
72	C	20	1.2	2.3	8	256	2.4	30	90	74	3	42
73	C	20	1.2	2.3	8	256	2.2	30	90	64	4	38
74	C	20	1.2	2.3	8	256	2.4	30	90	74	3	42
75	C	20	1.3	2.3	9	256	2.3	30	90	64	3	40
76	C	20	1.3	2.3	9	256	2.2	30	90	64	3	38
77	C	20	1.3	2.3	9	256	2.5	30	90	64	3	42
78	C	20	1.3	2.3	9	256	2.2	30	90	74	3	38
79	C	20	1.3	2.3	9	256	2.2	30	90	74	3	38
80	C	20	1.3	2.3	9	256	2.2	30	90	64	4	38
81	C	20	1.3	2.3	9	256	2.2	30	90	64	3	38
82	C	20	1.3	2.3	9	256	2.2	30	90	74	3	38

83	C	20	1.3	2.3	9	256	2.2	30	90	74	3	38
84	C	20	1.3	2.3	9	256	2.2	30	90	74	3	38
85	C	20	1.3	2.3	9	256	2.2	30	90	74	3	38
86	C	20	1.3	2.3	9	256	2.2	30	90	74	3	38
87	C	20	1.3	2.3	9	256	2.6	30	90	74	3	44
88	C	20	1.3	2.3	9	256	2.2	30	90	74	3	38
89	C	20	1.3	2.3	9	256	2.2	30	90	64	4	38
90	C	20	1.3	2.3	9	256	2.2	30	90	74	3	38
91	C	20	1.3	2.3	9	256	2.2	30	90	74	3	38
92	C	20	1.3	2.3	9	256	2.2	30	90	74	3	38
93	C	20	1.3	2.3	9	256	2.2	30	90	74	3	38
94	C	20	1.3	2.3	9	256	2.2	30	90	64	3	38
95	C	20	1.3	2.3	9	256	2.2	30	90	74	3	38
96	C	20	1.3	2.3	9	256	2.2	30	90	74	3	38
97	C	20	1.3	2.3	9	256	2.2	30	90	74	3	38
98	C	20	1.3	2.3	9	256	2.2	30	90	74	3	38
99	C	20	1.3	2.3	9	256	2.2	30	90	74	3	38
100	C	20	1.3	2.3	9	256	2.2	30	90	64	3	38
101	C	20	1.3	2.3	9	256	2.4	30	90	74	3	42
102	C	20	1.3	2.3	9	256	2.2	30	90	74	3	38
103	C	20	1.3	2.3	9	256	2.2	30	90	74	3	38
104	C	20	1.3	2.3	9	256	2.6	30	90	74	3	45
105	C	20	1.3	2.3	9	256	2.2	30	90	74	3	38
106	C	20	1.3	2.3	9	256	2.2	30	90	64	3	38
107	C	20	1.3	2.3	9	240	2.2	30	90	64	4	38
108	C	20	1.3	2.3	9	256	2.2	30	90	74	3	38
109	C	20	1.3	2.3	9	256	2.2	30	90	74	3	38
110	C	20	1.3	2.3	9	256	2.2	30	90	64	4	38
111	C	20	1.3	2.3	9	256	2.2	30	90	74	3	38
112	C	20	1.3	2.3	9	256	2.2	30	90	74	3	38
113	C	20	1.3	2.3	9	256	2.2	30	90	74	3	38
114	C	20	1.3	2.3	9	256	2.2	30	90	74	3	38
115	C	20	1.3	2.3	9	256	2.6	30	90	74	3	44
116	C	20	1.3	2.3	9	256	2.2	30	90	74	3	38
117	C	20	1.3	2.3	9	256	2.2	30	90	74	3	38
118	C	20	1.3	2.3	9	256	2.2	30	90	74	3	38
119	C	20	1.3	2.3	9	256	2.2	30	90	74	3	38
120	C	20	1.3	2.3	9	256	2.2	30	90	64	3	38
121	C	20	1.3	2.3	9	256	2.2	30	90	74	3	38
122	C	20	1.3	2.3	9	256	2.2	30	90	74	3	38
123	C	20	1.3	2.3	9	256	2.2	30	90	74	3	38
124	C	20	1.3	2.3	9	256	2.2	30	90	74	3	38
125	C	20	1.3	2.3	9	216	2.2	30	90	74	3	38
126	C	20	1.3	2.3	9	216	2.2	30	90	64	4	38

127	C	20	1.3	2.3	9	256	2.6	30	90	74	3	44
128	C	20	1.3	2.3	9	256	2.2	30	90	74	3	38
129	C	20	1.3	2.3	9	256	2.2	30	90	74	3	38
130	C	20	1.3	2.3	9	256	2.2	30	90	64	4	38
131	C	20	1.3	2.3	9	256	2.4	30	90	74	3	42
132	C	20	1.3	2.3	9	256	2.2	30	90	74	3	38
133	C	20	1.3	2.3	9	256	2.2	30	90	64	4	38
134	C	20	1.3	2.3	9	256	2.2	30	90	74	3	38
135	C	20	1.3	2.3	9	256	2.2	30	90	74	3	38
136	C	20	1.3	2.3	9	256	2.2	30	90	74	3	38
137	C	20	1.3	2.3	9	256	2.2	30	90	64	4	38
138	C	20	1.3	2.3	9	256	2.2	30	90	74	3	38
139	C	20	1.3	2.3	9	256	2.8	30	90	74	3	48
140	C	20	1.3	2.3	9	256	2.2	30	90	74	3	38
141	C	20	1.3	2.3	9	256	2.2	30	90	74	3	38
142	C	20	1.3	2.3	9	256	2.2	30	90	74	3	38
143	C	20	1.3	2.3	9	256	2.2	30	90	74	3	38
144	C	20	1.3	2.3	9	256	2.2	30	90	74	3	38
145	C	20	1.3	2.3	9	208	2.2	30	90	74	3	38
146	C	20	1.3	2.3	9	256	2.2	30	90	64	3	38
147	C	20	1.3	2.3	9	256	2.2	30	90	74	3	38
148	C	20	1.3	2.3	9	256	2.2	30	90	74	3	38
149	C	20	1.3	2.3	9	256	2.2	30	90	74	3	38
150	C	20	1.3	2.3	9	256	2.2	30	90	64	4	38
151	C	20	1.2	2.3	8	256	2.4	30	90	74	3	42
152	C	20	1.3	2.3	9	256	2.2	30	90	74	3	38
153	C	20	1.3	2.3	9	256	2.2	30	90	74	3	38
154	C	20	1.3	2.3	9	256	2.2	30	90	74	3	38
155	C	20	1.3	2.3	9	256	2.2	30	90	74	3	38

[a] All T1w voxel size = 1mm^3
[b] P = patient, C = control
[c] Repetition time (TR) expressed in units of ms
[d] Echo time (TE) expressed in units of 10^{-3} ms

Anatomical MRI pre-processing

Each participant's T1-weighted (T1w) image was corrected for intensity non-uniformity with 'N4BiasFieldCorrection'[1] (ANTs 2.3.3[2]) and was used as the T1w-reference. The T1w-reference was then skull-stripped with the 'antsBrainExtraction.sh' workflow using OASIS30ANTs as a target template. Brain tissue segmentation of cerebrospinal fluid, white matter, and gray matter was performed on the brain-extracted T1w using FSL's 'fast' tool. Volume-based spatial normalization to standard space (MNI152NLin2009cAsym) was performed through nonlinear registration with 'antsRegistration', using brain extracted versions of both T1w

reference and the T1w template. The ICBM 152 Nonlinear Asymmetrical template version 2009c was selected for spatial normalization.

Functional MRI pre-processing

For each run of fMRI data, a reference volume and its skull-stripped version were generated using fMRIPrep.[3] Head-motion parameters with respect to the BOLD reference (transformation matrices, and six corresponding rotation and translation parameters) were estimated before spatiotemporal filtering using 'MCFLIRT' from FSL. BOLD runs were slice-time corrected using '3dTshift' from AFNI. The BOLD time-series were then resampled into their original, native space by applying the transforms to correct for head-motion. The BOLD reference was then co-registered to the T1w reference using 'mri_coreg' (FreeSurfer) followed by FSL's FLIRT tool with the boundary-based registration cost-function. Co-registration was configured with twelve degrees of freedom to account for distortions remaining in the BOLD reference. The BOLD time-series were resampled into standard space, generating a pre-processed BOLD run in MNI152NLin2009cAsym space.

In order to remove signal variance in the time series associated with noise that can contaminate functional connectivity analyses,[4,5] several potential confounding time-series were calculated based on the pre-processed BOLD data: framewise displacement (FD), DVARS, and three global signals for the Cerebral Spinal Fluid (CSF), White Matter (WM), and the Grey Matter (GM) were computed. FD was computed using two formulations following Power[6] (absolute sum of relative motions, power_fd_dvars) and Jenkinson[7] (relative root mean square displacement between affines, mcflirt). Additionally, a set of physiological regressors were extracted to allow for component-based noise correction ('CompCor'). Principal components were estimated after high-pass filtering the preprocessed BOLD time-series (using a discrete cosine filter with 128s cut-off) for the two 'CompCor' variants: temporal (tCompCor) and anatomical (aCompCor). tCompCor components are then calculated from the top 2% variable voxels within the brain mask. For aCompCor, three probabilistic masks (CSF, WM, and combined CSF+WM) were generated in anatomical space. Finally, these masks are resampled into BOLD space and binarized by thresholding at 0.99. Components were also calculated separately within the WM and CSF masks. For each CompCor decomposition, the *k* components with the largest singular values were retained, such that the retained components' time series are sufficient to explain 50 percent of

variance across the nuisance mask (CSF, WM, combined, or temporal). The remaining components are dropped from consideration.

fMRIPrep produces a confounds file with the factors described above but does not perform the actual denoising of the BOLD data during pre-processing (the parameters included in the denoising pipeline discussed below). The head-motion estimates calculated during that preprocessing step were also placed within the corresponding confounds file. The confound time series derived from head motion estimates and global signals were expanded with the inclusion of temporal derivatives and quadratic terms for each, enabling a 36-parameter denoising strategy.[8] Frames that exceeded a threshold of 0.5 mm FD or 1.5 standardized DVARS were annotated as motion outliers.

The pre-processed resting fMRI data outputted by fMRIPrep was analyzed using CONN[9] (release 22.a)[10] and SPM[11] (release 12.7771). Functional voxels were interpolated to 2x2x2mm^3 and smoothed using spatial convolution with a Gaussian kernel of 4 mm full-width half maximum (FWHM). Using the confound file created by fMRIPrep as described above, functional data were denoised using a standard pipeline[12] that includes the regression of potential confounding effects characterized by white matter timeseries (5 CompCor noise components), CSF timeseries (5 CompCor noise components), motion parameters (6 factors),[13] outlier scans (below 129 and 147 factors for controls and patients, respectively),[6] session effects and their first order derivatives (2 factors), QC_cosine regressors (7 and 5 components for controls and patients, respectively), and linear trends (2 factors) within each functional run, followed by bandpass frequency filtering of the BOLD timeseries between 0.008 Hz and 0.09 Hz.[14] From the number of noise terms included in this denoising strategy, the effective degrees of freedom of the BOLD signal after denoising were estimated to range from 12.3 to 192.7 (average 70.5) across the glioma cohort and 16.2 to 72.9 (average 44.8) across the neurotypical cohort.[15] All hypothesis testing treated subject as a random factor.

Subject-level normalization of functional connectivity

Functional connectivity (FC) is represented as a Fisher-transformed bivariate correlation coefficient from a weighted general linear model, defined separately for each ROI-to-ROI pair (ROI = region-of-interest). For every participant (patient and controls), we computed ROI-to-ROI FC among all right-hemisphere ROIs in the Yan homotopic atlas.[16] The resulting right-hemisphere connectivity matrix was mean-normalized at the subject-level, resulting in a ROI-to-ROI

connectivity matrix of z-scores (RRCz). This facilitates comparison across participants and focuses analyses on the variance in FC across the sample.[17] Additionally, it accounts for magnitude differences in BOLD values across patients and controls that might result from inter-scanner differences—analyses are based on the patterns of variance in connectivity among ROIs, and how those patterns relates to characteristics of patients' tumors.

To test whether subject-level normalization of functional connectivity matrices was sufficient to control for inter-scanner differences, we first compared the distribution of non-standardized functional connectivity (Supplementary Figure 9A) and connectivity normalized at the subject level (Supplementary Figure 9B) across all right-hemisphere edges between patients and controls. We used Wilcoxon rank-sum tests to test whether there were significant differences in center, spread, and shape of the distributions.[18,19] We found a significant difference in the non-standardized connectivity distributions ($z = 9.38$, $p = 6.36\text{E-}21$, two-tailed Wilcoxon rank-sum test with approximate p-value), such that patients exhibit slightly stronger connectivity on average ($\bar{x}_{\text{patients}} = 0.138$, $\bar{x}_{\text{controls}} = 0.130$). This could be the result of scanner differences or changes intrinsic to glioma patients (e.g., functional reorganization). However, subject-level normalization abolished the differences in the overall distributions of connectivity ($z = -1.05$, $p = 0.30$, two-tailed Wilcoxon rank-sum test with approximate p-value). These analyses indicate that subject-level normalization is sufficient to control for systematic shifts in the connectivity distribution and focuses the analysis on differences in relative edge strength.

Support Vector Machine

General Principles

A linear kernel was chosen for all support vector machines (SVMs) given the high-dimensional nature of our imaging data. One key benefit of linear kernels is the reduced risk of overfitting,[20] which is a potential concern for high dimensional data. For this reason, many prior neuroimaging studies opt for a linear kernel.[21,22] Importantly, an initial pilot test that allowed kernel selection during hyperparameter tuning also favored the linear kernel, suggesting that the data were linearly separable. The strong classification performance of the participant identity and the *IDH*-mutation analyses provides further evidence for lack of overfitting and linear separation. To avoid overfitting and ensure unbiased model evaluation, we implemented nested cross-validation to optimize and train our SVMs. We used feature selection for patient-only analyses, which we established to be critical for model stability and optimal performance given the low

sample size relative to the participant-only analysis (n = 48 vs 155). We used parallelization for all hyperparameter optimization. We used permutation testing to assess performance of all models against chance.

Participant Identity

We aimed to classify patients versus healthy controls using resting-state functional connectivity data derived from the right hemisphere. To preserve the subtle, spatially distributed connectivity patterns hypothesized to underlie group differences, we used the full feature space without dimensionality reduction. Classification with nested cross-validation was performed using an SVM learner implemented via MATLAB's 'fitclinear' function with a dual stochastic gradient descent (DSGD) solver and L2 (ridge) regularization. The combination of these parameters were selected intentionally, along with fitclinear, as they are optimized for accurately classifying high-dimensional datasets with minimal computation time (see fitclinear documentation: https://www.mathworks.com/help/stats/fitclinear.html). The strength of regularization (λ) was treated as a tunable hyperparameter and optimized using Bayesian optimization with an expected-improvement-plus acquisition function. Model performance was evaluated using a nested leave-one-out cross-validation (LOOCV) framework, where the inner loop (5-fold CV) was used for hyperparameter tuning (see Supplementary Figure 1). Specifically, the outer loop split the full dataset into a training and test set and the training set is fed into the inner loop for tuning. After determining the optimized hyperparameter in the inner loop, a final model was trained on the outer loop's training set using the identified regularization strength. To complete an iteration of the outer loop, a prediction is generated for the held-out test subject using the trained final model. Importantly, hyperparameter optimization was performed using *only* the external fold's training data, meaning that the parameters were naïve to the test set; this is a critical step in preventing data leakage. The outer loop is complete when all subjects have been held out as the test-subject and have an associated prediction.

Next, accuracy, precision, and recall are calculated using the formulas in Table 2, with patients considered the 'positive' case (note: the binary numerical value is treated functionally as a label. In other words, despite patients being labeled as 0 in the dataset, they are the positive cases). Across all analyses, the 'positive' cases refer to the group that we hypothesize to demonstrate more functional reorganization (patients, region-specific lesion presence, *IDH*-mutant, low-grade, and small lesions), which enables interpretation of model performance in line

with our hypotheses. Lastly, the entire pipeline is repeated 100 times to assess average model performance and model stability. Across these 100 iterations we found an average accuracy of 89% (s = 1%), average precision of 84% (s = 2%), and average recall of 79% (s = 3%). For comparison, Minimum Redundancy Maximum Relevance (MRMR) feature selection (the method used in other analyses, see below) was tested but resulted in reduced classification performance (accuracy = 84%, precision = 74%, recall = 76%), suggesting that using the full feature space yielded more optimal results.

Region-Specific Lesion Presence

A separate classification task was designed to predict the presence or absence of a lesion within specific regions-of-interest (ROIs). We first identified all left-hemisphere ROIs that had at least 20% of patient participants (i.e., $n \geq 10$) with lesions present to ensure a reasonable minimum class imbalance (1:4), resulting in 42 suprathreshold ROIs. We then applied the following pipeline to each ROI individually.

We used a nested cross-validation approach to optimize and train our SVM (see Supplementary Figure 1). Feature selection was conducted within each external fold using MRMR; this method identifies features that are strongly correlated with the outcome while minimizing correlations between features. We hypothesized that fewer features would be sufficient to classify lesion presence in specific ROIs; that is, we anticipated that distinct, spatially relevant connectivity features would emerge for each ROI (e.g., due to homotopic redistribution[23-25]) that could be diluted if all features were included. After applying MRMR, one must identify a reasonable threshold feature importance, which is determined by finding the 'elbow point' in plotting features ranked by their relative importance scores (see fscmrmr documentation: https://www.mathworks.com/help/stats/fscmrmr.html). Thresholds were identified independently for each ROI and ranged from 0.01 to 0.05, with 0.05 being the most common. While the ROI-specific threshold was determined using fscmrmr on the entire dataset (Train + Test), we re-applied feature selection independently for each external fold's training set (using the threshold defined on the entire dataset) and then selected the same subset of features for the test set. Because the threshold is defined on the entire dataset, it is theoretically possible that some external folds will not have any features that meet the threshold (and likely to be the case for the permutation test). To prevent creation of null models, the top five features were selected if no features were chosen after applying the threshold.

After the feature space was defined, we optimized and trained our model using nested cross-validation. Like the participant identity analysis, we used external LOOCV to evaluate model performance. However, due to the large number of models (as this pipeline was applied independently to each ROI), we chose internal LOOCV rather than to perform 100 iterations to reduce computational demands while still ensuring model stability. Since we applied feature reduction, we were able to use MATLAB's 'fitcsvm', which is appropriate for low-to-moderate dimensional data. We optimized the regularization parameter (for fitcsvm, this is the Box Constraint, or 'C') with the same Bayesian approach and acquisition function as before. We then trained a final model on the external fold's training set using the optimized Box Constraint. Lastly, we generated predictions for each external fold's test set and calculated model performance metrics using formulas in Table 2. Lesion presence was defined as the 'positive' class, such that recall is interpreted as the model's ability to correctly classify subjects with lesion present in a given ROI.

Using an integrative measure is critical for effectively comparing classifier performance across ROIs with varying degrees of class imbalance. In addition to accuracy, precision, and recall, we calculated the Matthews correlation coefficient (MCC = $\frac{TP \times TN - FP \times FN}{\sqrt{(TP+FP)(TP+FN)(TN+FP)(TN+FN)}}$, where TP = true positives, TN = true negatives, FP = false positives, and FN = false negatives) as a unified metric.[26] MCC is widely regarded as a robust, overall assessment of binary classifier performance, particularly for imbalanced datasets.[27,28] Unlike other metrics, it accounts the entire confusion matrix, is invariant to the labeling of the positive class, and generates a high score only if the classifier can correctly predict the majority of positive cases and the majority of negative cases.[27,29] MCC values were computed for each ROI-specific SVM, as well as for each iteration of the permutation tests, using the formula above. We used a two-stage Monte Carlo permutation testing approach: an initial 100-iteration screen to identify candidate ROIs based on significant MCC values, then a 1000-iteration permutation test for all candidate ROIs ($p_{\mathrm{MCC}} < 0.05$ on the 100-iteration screen). This decision was guided by the extreme computational expense required to do 1000-iterations for each of the 42 ROI-specific SVMs.

Tumor Characteristics

We next sought to test whether the right connectivity matrix could predict tumor characteristics (*IDH*-mutation, WHO grade, and tumor size). For these tasks, MRMR feature selection was again applied within each external fold, using a 0.05 importance threshold (or selecting the top five features when the threshold yielded none). In the inner loop, we then trained

SVM models using MATLAB's fitcsvm function, tuning the Box Constraint hyperparameter (C) using Bayesian optimization. Here, the nested cross-validation approach was identical to the participant identity analysis, such that external LOOCV was used for model performance evaluation and internal 5-fold cross-validation was used to tune the regularization parameter. After completion of the outer loop, accuracy, precision, and recall are calculated using the formulas in Table 2. Across all analyses, the 'positive' cases refer to the group that we hypothesize to demonstrate more functional reorganization (*IDH*-mutant, low-grade, and small lesions), which enables interpretation of model performance in line with our hypotheses. Lastly, the entire pipeline is repeated 100 times to assess average model performance and model stability. We performed permutation testing for each classification problem using 1000 iterations.

For comparison, we also performed this exact pipeline without feature selection. We found that feature selection was critical for both model stability and model performance. This likely reflects the difference in sample size between these analyses and the participant identity analysis.

Supplementary Table 2. Summary of Support Vector Machine Approaches Across Analyses

Parameter	Participant identity	Lesion Presence	Tumor Characteristics
**Kernel**	Linear	Linear	Linear
**External cross-validation**	LOOCV[a]	LOOCV	LOOCV
**Internal cross-validation**	5-fold	LOOCV	5-fold
**Model stability mechanism**	100 iterations	Internal LOOCV	100 iterations
**Feature selection**	—	MRMR[b]	MRMR
**MATLAB function**	fitclinear	fitcsvm	fitcsvm
**Solver**	DSGD[c]	SMO[d]	SMO[d]
**Regularization type**	Ridge (L2)	—	—
**Regularization parameter**	λ	Box Constraint (C)	Box constraint (C)
**Optimization**	Bayesian	Bayesian	Bayesian
**Acquisition function**	Expected-improvement-plus	Expected-improvement-plus	Expected-improvement-plus
**Permutation test**	1000 iterations	Two-stage (100 ⇒ 1000)	1000 iterations

[a] Leave-one-out cross-validation
[b] Minimum Redundancy Maximal Relevance
[c] Dual stochastic gradient descent
[d] Sequential Minimal Optimization

Anatomical clustering of lesions drives unique pattern of contralesional functional connectivity

To assess whether the differences in lesion location between the patient clusters is statistically significant, we conducted voxel-wise Barnard's exact tests comparing lesion overlap

between patient clusters. For each left-hemisphere voxel with at least one lesion present, we counted the subjects with lesion overlap for each cluster. For each voxel, we constructed a 2×2 contingency table comparing lesion overlap between the two clusters and applied Barnard's exact test. To ensure consistency with the difference map shown in Figure 3C and to account for the unequal sample sizes between clusters, we calculated the No Overlap value by subtracting Overlap values from each cluster's maximum observed overlap rather than total cluster sample size. Specifically, the highest lesion overlap observed in cluster one was $n = 6$, and in cluster two was $n = 10$. Using these values avoids inflating differences due to the overall sample size imbalance ($n = 9$ vs. $n = 39$, respectively).

Given the relatively small sample size and broad coverage of the left hemisphere (i.e., many comparisons), no voxels survived correction for multiple comparisons using False Discovery Rate. As an exploratory analysis, we generated a voxel-wise uncorrected *p*-value map and applied a threshold at alpha = 0.05.

Supplementary Results

The presence of a left-hemisphere glioma is predicted by right-hemispheric functional connectivity

We explored whether lesion volume, age, *IDH*-mutation, and WHO grade are significantly associated with misclassification. Out of the 100 iterations, we identified an exemplar trial whose performance (in particular, recall) closely matched that of average performance (79% recall). We found no significant difference in lesion size between patients misclassified and patients correctly classified ($z = -0.42$, $p = 0.68$, two-tailed Wilcoxon rank-sum test with approximate *p*-value). The relationship between lesion size and likelihood of inducing contralesional changes is complex. On the one hand, patients with larger lesions may have more aggressive tumors with greater momentum, which are less likely to cause functional reorganization. On the other hand, patients with very small tumors may also be less likely to recruit the contralesional hemisphere and rather may employ one of the other patterns of reorganization (persistence of function within the tumor, perilesional reorganization, or more distally within the ipsilesional hemisphere). We also found no significant difference in age at the time of scan between these groups of patients ($z = 0.33$, $p = 0.74$, two-tailed Wilcoxon rank-sum test with approximate *p*-value).

For categorical variables such as *IDH*-mutation and WHO grade, we instead constructed contingency tables of classification (hit or miss) vs i) *IDH*-mutation and ii) WHO grade and performed Barnard's tests to assess for significance (Supplementary Table 2). Barnard's test is an exact test similar to Fisher's exact test; however, it is often more powerful for 2 x 2 contingency tables and applicable in circumstances where both margins are not fixed[30]. We found that neither *IDH*-mutation status nor WHO grade was significantly associated with misclassification (p = 0.48 and 0.45, respectively, for Barnard's exact tests on the contingency tables below). We hypothesize that replication of this study in a larger cohort may yield associations with these clinical variables, but we did not observe evidence in our sample.

Supplementary Table 3. Tumor Characteristics and Binary Classification Performance

**Classification[a]**	***IDH*-mutant**	***IDH*-wildtype**
**Hit**	20	18
**Miss**	5	5
	**Low-grade**	**High-grade**
**Hit**	13	25
**Miss**	3	7

[a] Classification of patients: Hit = patient correctly classified, Miss = patient misclassified as control

Supplementary Figures

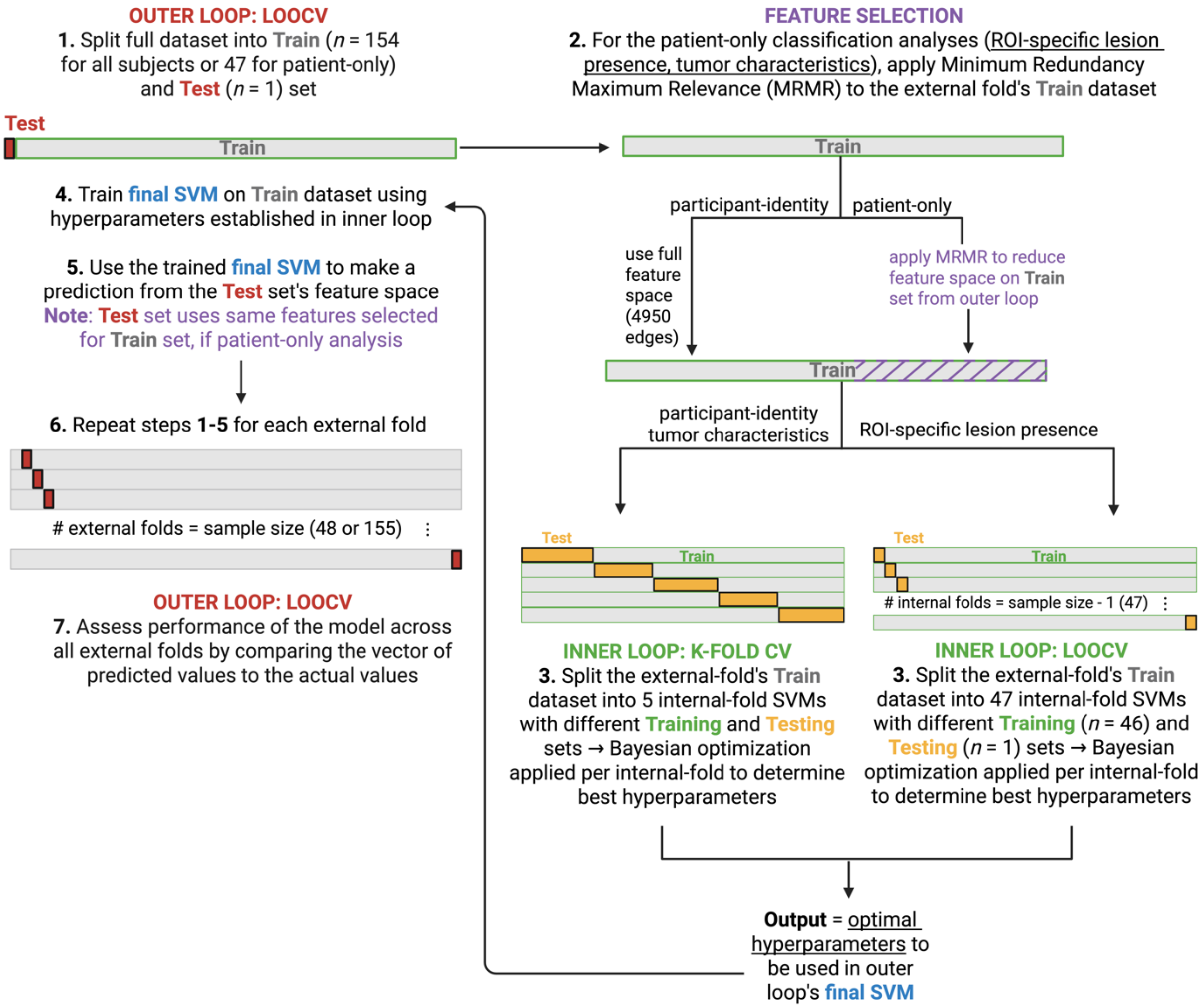

Supplementary Figure 1. Nested cross-validation support vector machine framework. Leave-one-out cross-validation (outer loop) was used to evaluate model performance for all support vector machine analyses. First, the outer loop splits the full dataset into training and testing data for a given external fold. Within the inner loop, feature selection using Minimal Redundancy Maximal Relevance (MRMR) was applied only in patient-only models (region-of-interest (ROI) specific lesion presence, tumor characteristics – *IDH*-mutation, WHO grade, tumor size). Next, internal cross-validation was applied to tune the regularization parameter (C or λ) on the external fold's training set. Specifically, 5-fold internal-cross-validation was used for participant identity and tumor characteristics analyses and leave-one-out cross-validation was used for the ROI-specific analysis. The optimal hyperparameter from the inner loop was used to train the outer loop model on the external fold's training set. Lastly, the trained model generates a prediction from the unseen external fold's testing data. For the participant identity analysis, the sample size is 155 participants. All other analyses are conducted on the sample of patients only (n = 48). Created in BioRender. Strawderman, E. (2025) https://BioRender.com/5bcuvdn.

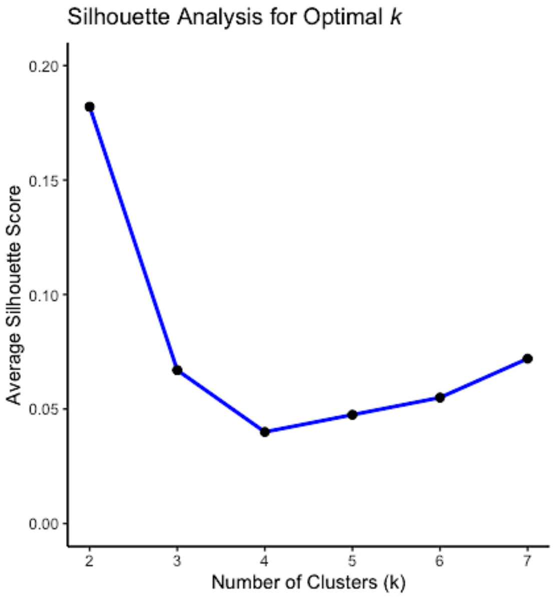

Supplementary Figure 2. Silhouette analysis reveals optimal value of two for *k*-means clustering of the all-patient connectivity matrix.

We performed a silhouette analysis to determine the optimal *k* for *k*-means clustering. The silhouette coefficient is calculated by the formula: (separation - cohesion) / maximum(separation, cohesion). For values of *k* = 2 through 7, we performed *k*-means clustering on the all patient right-hemispheric connectivity matrix (RRCz). For each value of *k*, the *k*-means clustering algorithm performed 1000 independent replicates to avoid local minima. We used the 'silhouette' function in MATLAB to calculate the average silhouette coefficient for each *k*-means clustering solution. We chose the value of *k* and the associated cluster assignments with the highest silhouette coefficient (*k* = 2 with average silhouette score of 0.18).

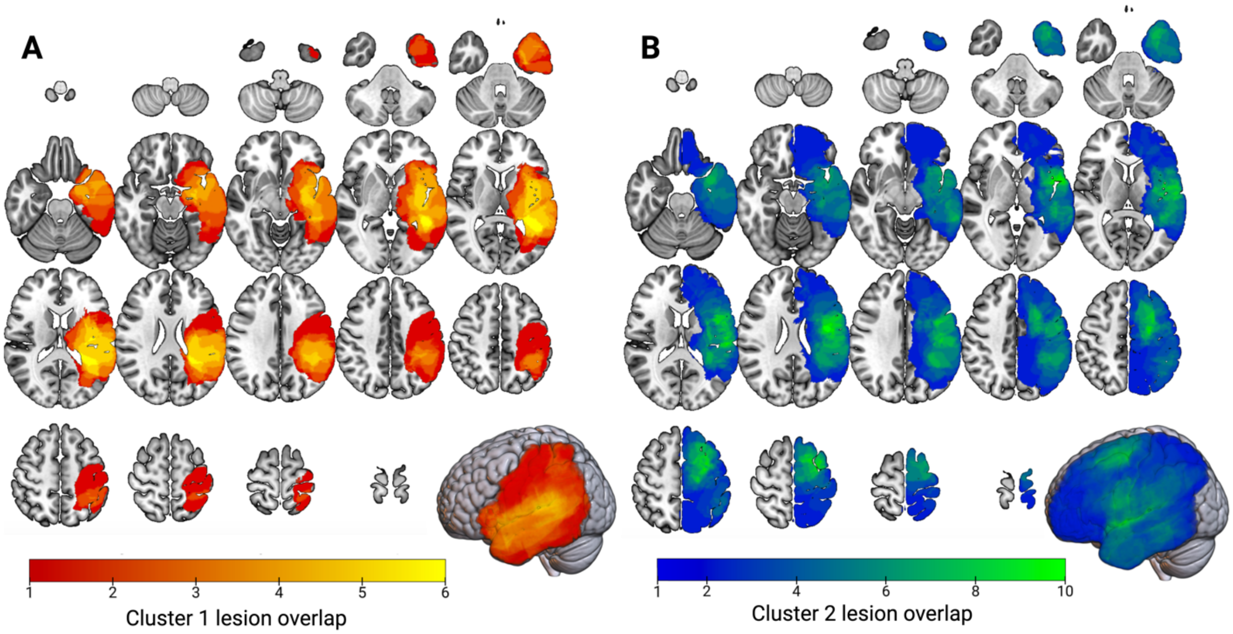

Supplementary Figure 3. Patient clusters form contiguous lesion overlap maps with distinct spatial foci.

After generating the cluster assignments from the optimal *k*-means clustering algorithm, we then constructed lesion overlap maps for all the subjects within cluster one **(A)** and cluster two **(B).** The above overlap maps are unthresholded (i.e., displaying all subject lesions ranging from 1 subject to their respective number of maximal overlap). Created in BioRender. Strawderman, E. (2025) https://BioRender.com/fltlrxk.

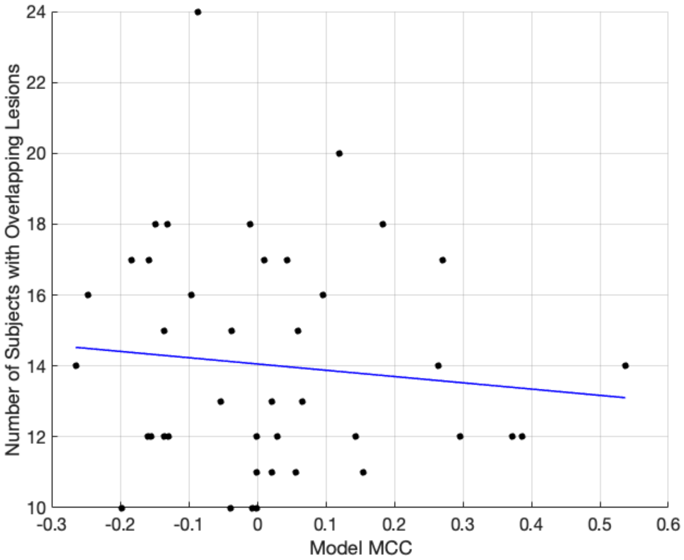

Supplementary Figure 4. Model performance is not associated with degree of class imbalance across ROIs.

The number of subjects with a lesion present—a marker of class imbalance—was not significantly associated with overall model performance as measured by Matthew's correlation coefficient (MCC) across the 42 suprathreshold ROIs (Spearman ρ_{40} = -0.069; p =0.66). Note that MCC values range from -1 (worst) to 1 (perfect), with zero representing random guessing, making its interpretation comparable to that of a standard Pearson correlation.

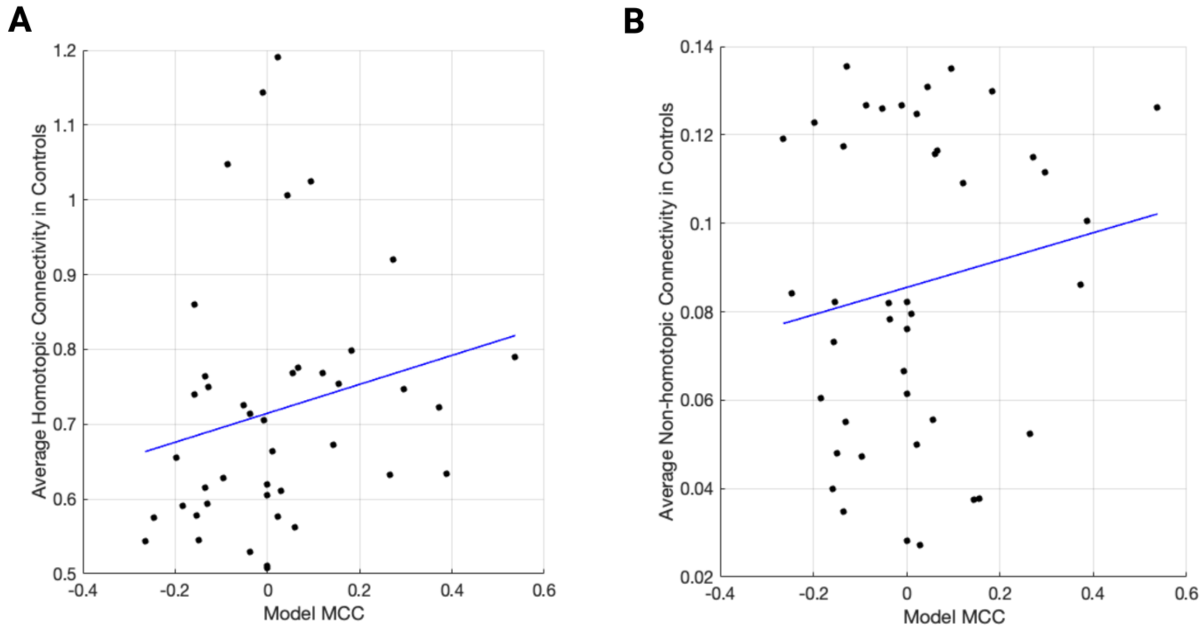

Supplementary Figure 5. ROI model performance is associated with the strength of homotopic connectivity.

(A) Scatter plot between ROI-specific model performance (Matthew's correlation coefficient, or MCC) and average homotopic connectivity across 42 suprathreshold ROIs (Spearman $\rho_{40} = 0.35$; $p = 0.022$). Homotopic connectivity is defined as functional connectivity between a given ROI and its mirror image. **(B)** Scatter plot between ROI-specific model performance (MCC) and the average functional connectivity to the 99 non-homotopic ROIs across all 42 suprathreshold ROIs (Spearman $\rho_{40} = 0.11$; $p = 0.49$). For both **(A)** and **(B)**, the average homotopic and non-homotopic connectivity values were calculated in the cohort of age-matched healthy controls ($n = 107$) for each individual suprathreshold ROI. MCC ranges from -1 to 1 and provides a balanced measure of binary classification performance, accounting for all confusion matrix elements and is robust to class imbalance. Degrees of freedom for the two-tailed Spearman rank correlations are reported as subscripts to the rho symbol above. Created in BioRender. Strawderman, E. (2025) https://BioRender.com/vr9wu78.

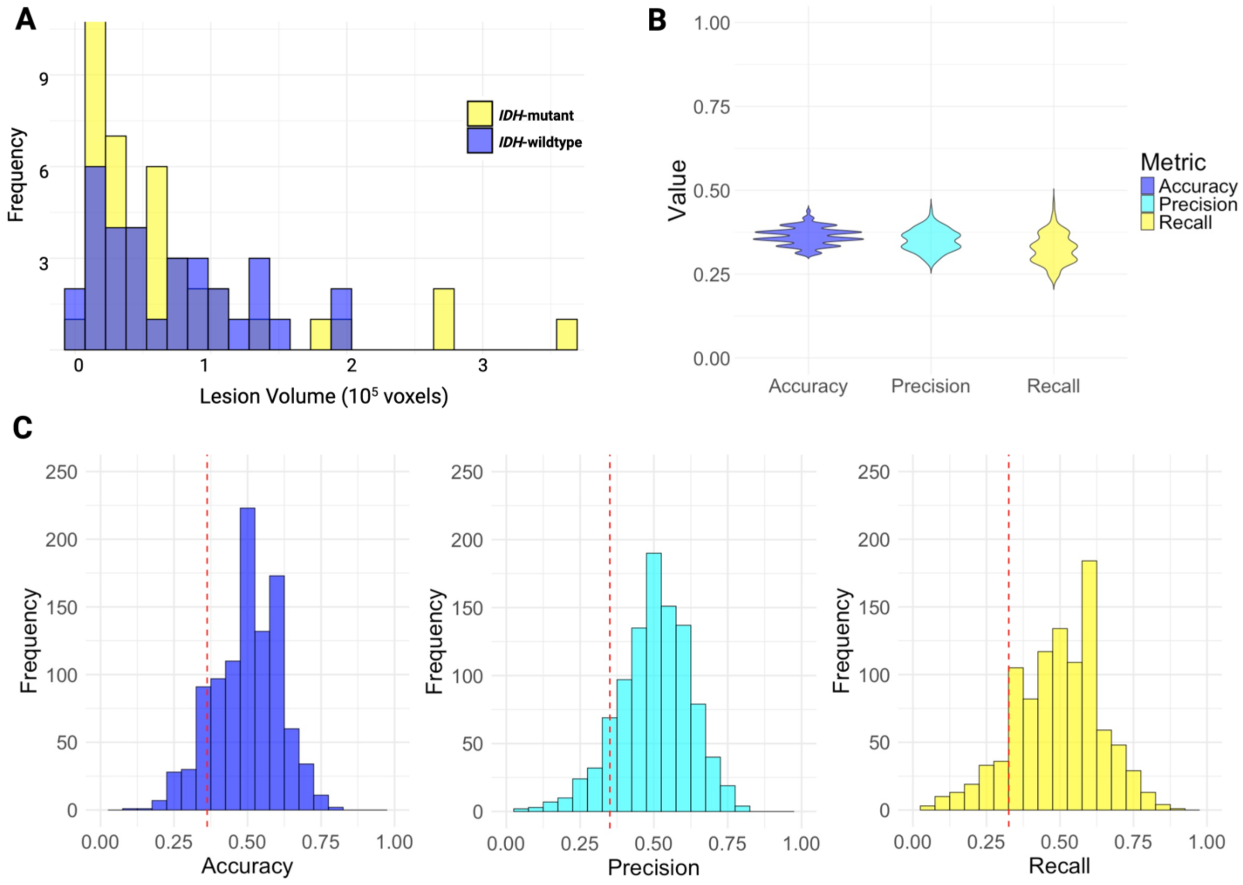

Supplementary Figure 6. Right-hemisphere connectivity matrix does not predict lesion size. (A) Overlapping histograms of lesion volume as measured in 10^5 voxels. Tumor volume in *IDH*-mutant and *IDH*-wildtype patients were not significantly different ($z = -0.01$, $p = 0.99$, two-tailed Wilcoxon rank-sum test with approximated p-value). **(B)** Violin plots of model performance across 100 iterations (accuracy: $\bar{x} = 0.36$, $s = 0.03$; precision: $\bar{x} = 0.35$, $s = 0.03$; recall: $\bar{x} = 0.33$, $s = 0.04$). Patients with small lesions were the 'positive' class. A lesion was classified as small if its volume was in the bottom 50% of all patients' lesion volumes and large if the volume was in the top 50%. **(C)** Distributions of accuracy ($\bar{x} = 0.50$, $s = 0.11$, $p = 0.87$), precision ($\bar{x} = 0.50$, $s = 0.12$, $p = 0.89$), and recall ($\bar{x} = 0.50$, $s = 0.15$, $p = 0.89$) generated by permutation tests. The vertical red lines indicate the mean values from Supplementary Figure 6B. Empirical p-values are determined from comparison of the mean model performance metric and the associated Monte-Carlo permutation distribution using the formula $p = \frac{r+1}{n+1}$, where r is the number of iterations ≥ mean model performance value and n = number of total iterations ($n = 1000$). For **(B)** and **(C)**, distributions are shown on scale of possible model performance metrics (0 to 1) to facilitate assessment of model stability. For **(A)** – **(C)**, the sample consisted of 48 glioma patients ($n_{large} = 24$, $n_{small} = 24$). Created in BioRender. Strawderman, E. (2025) https://BioRender.com/ot4kbtx.

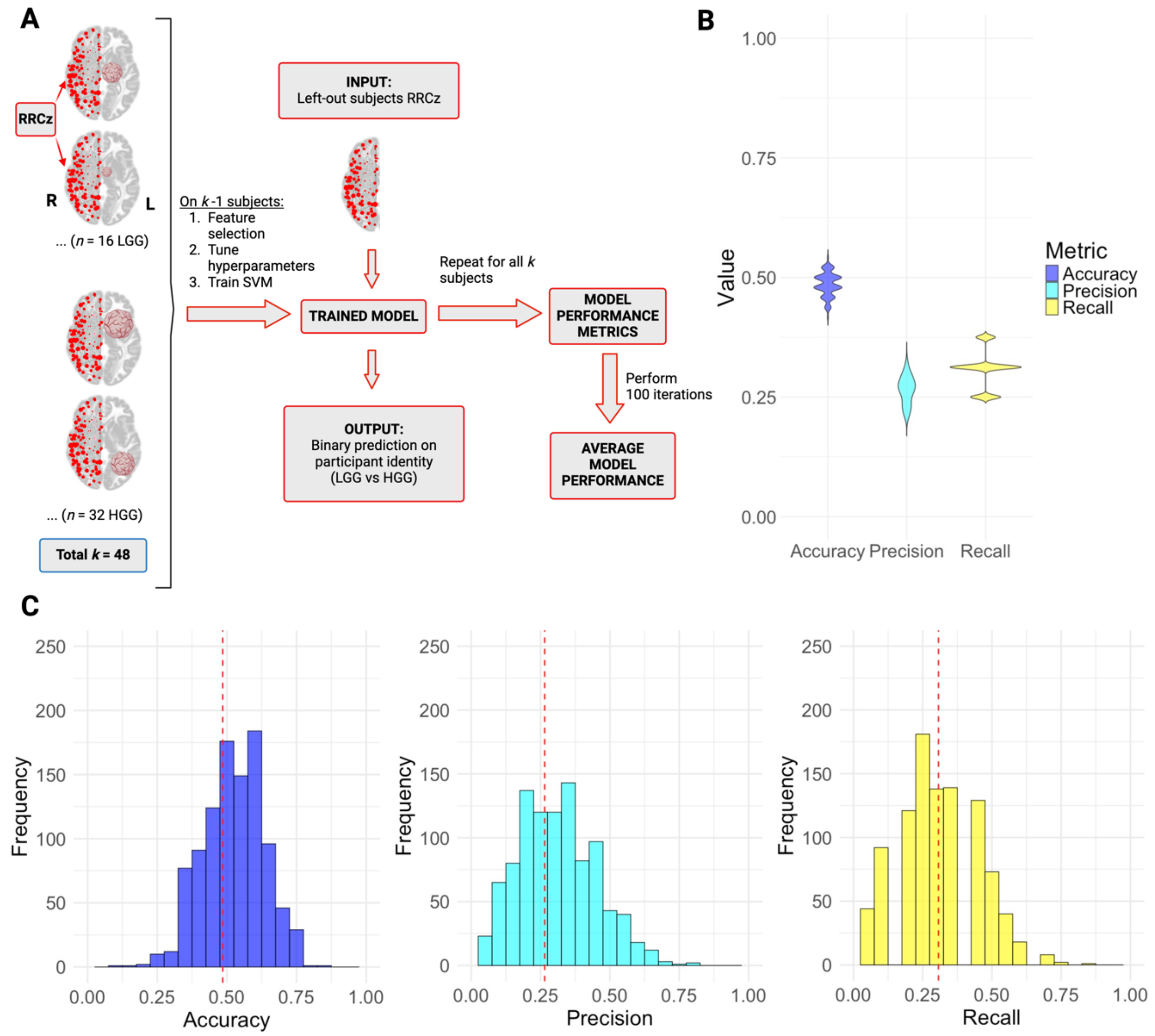

Supplementary Figure 7. Right-hemisphere connectivity matrix does not predict tumor grade.
(A) The same support vector machine (SVM) pipeline used for *IDH*-mutation and size was applied to classify patients by WHO grade. Patients with low-grade gliomas (LGGs) were the 'positive' class. **(B)** Violin plots of model performance across 100 iterations (accuracy: $\bar{x} = 0.49$, $s = 0.02$; precision: $\bar{x} = 0.26$, $s = 0.03$; recall: $\bar{x} = 0.31$, $s = 0.04$). **(C)** Distributions of accuracy ($\bar{x} = 0.53$, $s = 0.11$, $p = 0.63$), precision ($\bar{x} = 0.31$, $s = 0.14$, $p = 0.58$), and recall ($\bar{x} = 0.31$, $s = 0.15$, $p = 0.55$) generated by permutation tests. The vertical red lines indicate the mean values from Supplementary Figure 7B. Empirical p-values are determined from comparison of the mean model performance metric and the associated Monte-Carlo permutation distribution using the formula $p = \frac{r+1}{n+1}$, where r is the number of iterations ≥ mean model performance value and n = number of total iterations ($n = 1000$). For **(B)** and **(C)**, distributions are shown on scale of possible model performance metrics (0 to 1) to facilitate assessment of model stability. For **(A)** – **(C)**, the sample consisted of 48 glioma patients ($n_{HGG} = 32$, $n_{LGG} = 16$). Created in BioRender. Strawderman, E. (2025) https://BioRender.com/6b0tvm8.

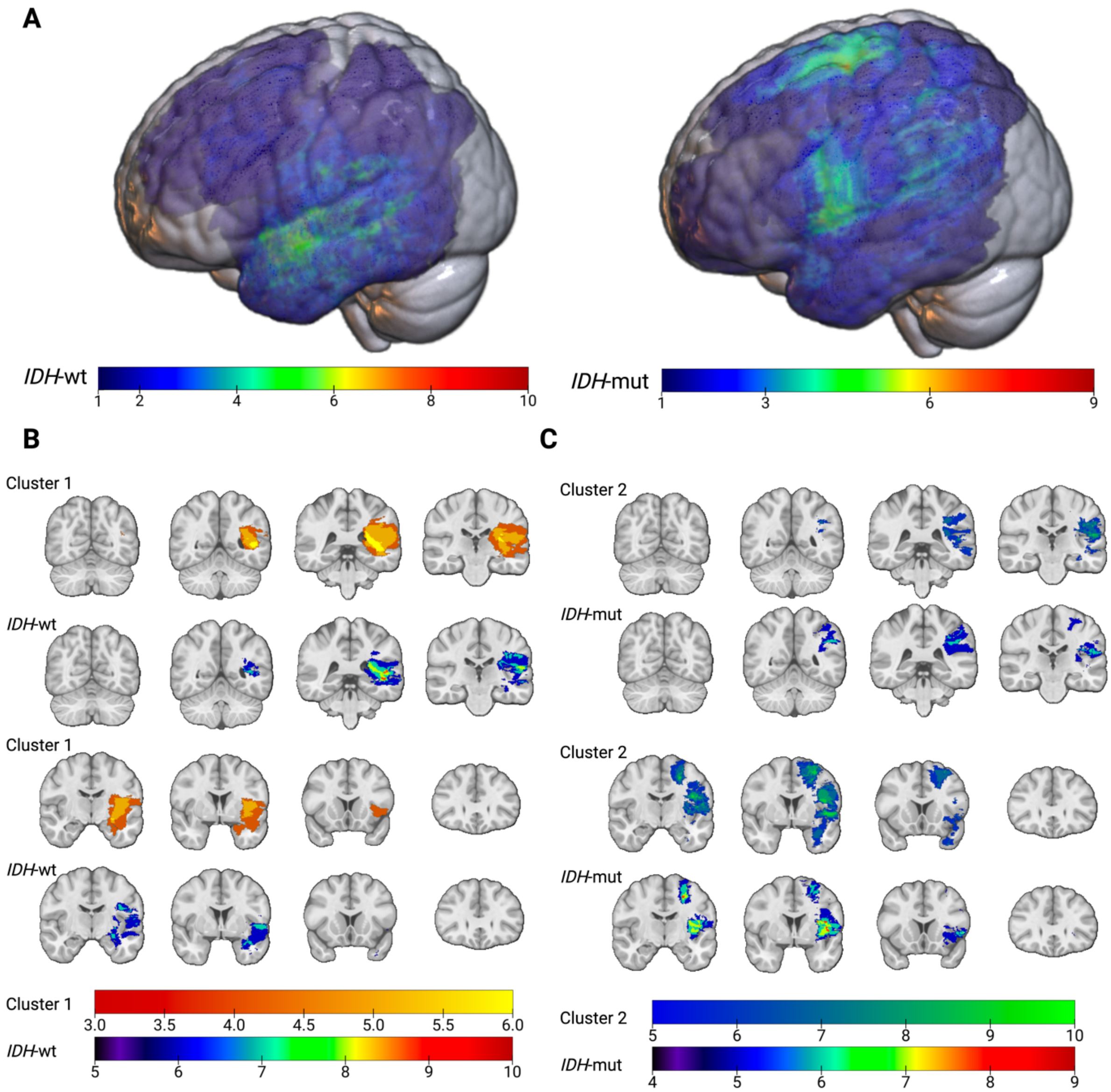

Supplementary Figure 8. *IDH*-mutation is associated with lesion location and cluster identity.

(A) Lesion overlap maps for *IDH*-wildtype and *IDH*-mutant lesions. The peak of *IDH*-wildtype lesions is in the posterior perisylvian region. The highest concentrations of *IDH*-mutant lesions are in the pars opercularis, superior frontal gyrus, and insula. Color maps demonstrate maximum overlap: $n_{IDH\text{-mut}} = 9$, $n_{IDH\text{-wt}} = 10$. **(B)** Peak lesion overlap map for cluster one and *IDH*-wildtype lesions, coronal view. **(C)** Peak lesion overlap map for cluster two and *IDH*-mutant lesions, coronal view. Both maps are thresholded to display the top half of values to emphasize spatial foci. For **(B)** and **(C)**, both maps are thresholded to display the top half of values to emphasize spatial foci, as in Fig. 3. The top two rows correspond to slices y = -60 through y = -18, and the bottom two rows correspond to slices y = -4 through y = 40. Created in BioRender. Strawderman, E. (2025) https://BioRender.com/r65mig8.

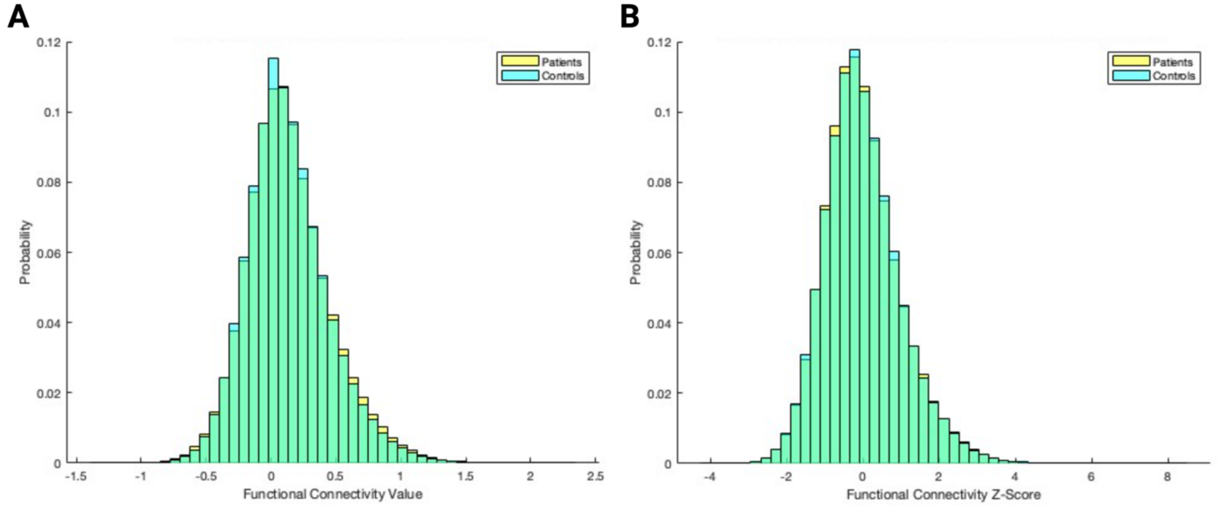

Supplementary Figure 9. Subject-level normalization of functional connectivity controls for overall differences in distribution between patients and controls.
(A) Distribution of non-standardized ROI-to-ROI functional connectivity values across all right hemisphere edges between patients (yellow, n = 237600 connectivity values) and controls (cyan, n = 529,650 connectivity values). Green = overlap in distributions. Non-standardized connectivity values differed significantly between groups (z = 9.38, p = 6.36E-21, two-tailed Wilcoxon rank-sum test with approximated p-value), with patients showing slightly higher average connectivity ($\bar{x}_{\text{patients}}$ = 0.138, $\bar{x}_{\text{controls}}$ = 0.130). **(B)** Distribution of subject-level normalized ROI-to-ROI functional connectivity across all right hemisphere edges between patients (yellow) and controls (cyan) in the right hemisphere. After subject-level normalization, no significant group difference remained (z = –1.05, p = 0.30, two-tailed Wilcoxon rank-sum test with approximated p-value). Created in BioRender. Strawderman, E. (2025) https://BioRender.com/7u9pjoz.

Supplementary References

1. Tustison NJ, Avants BB, Cook PA*, et al*. N4ITK: improved N3 bias correction. *IEEE transactions on medical imaging*. 2010;29(6):1310-1320.
2. Avants BB, Tustison NJ, Song G, Cook PA, Klein A, Gee JC. A reproducible evaluation of ANTs similarity metric performance in brain image registration. *Neuroimage*. 2011;54(3):2033-2044.
3. Esteban O, Markiewicz CJ, Blair RW*, et al*. fMRIPrep: a robust preprocessing pipeline for functional MRI. *Nat Methods*. Jan 2019;16(1):111-116. doi:10.1038/s41592-018-0235-4
4. Liu TT. Noise contributions to the fMRI signal: An overview. *NeuroImage*. 2016;143:141-151.
5. Murphy K, Birn RM, Bandettini PA. Resting-state fMRI confounds and cleanup. *Neuroimage*. 2013;80:349-359.
6. Power JD, Mitra A, Laumann TO, Snyder AZ, Schlaggar BL, Petersen SE. Methods to detect, characterize, and remove motion artifact in resting state fMRI. *Neuroimage*. 2014;84:320-341.
7. Jenkinson M, Bannister P, Brady M, Smith S. Improved Optimization for the Robust and Accurate Linear Registration and Motion Correction of Brain Images. *NeuroImage*. 2002/10/01/ 2002;17(2):825-841. doi:https://doi.org/10.1006/nimg.2002.1132
8. Satterthwaite TD, Elliott MA, Gerraty RT*, et al*. An improved framework for confound regression and filtering for control of motion artifact in the preprocessing of resting-state functional connectivity data. *Neuroimage*. 2013;64:240-256.
9. Whitfield-Gabrieli S, Nieto-Castanon A. Conn: a functional connectivity toolbox for correlated and anticorrelated brain networks. *Brain connectivity*. 2012;2(3):125-141.
10. Nieto-Castanon A, Whitfield-Gabrieli S. CONN functional connectivity toolbox (RRID: SCR_009550), Version 21. Hilbert Press; 2021.
11. Penny WD, Friston KJ, Ashburner JT, Kiebel SJ, Nichols TE. *Statistical parametric mapping: the analysis of functional brain images*. Elsevier; 2011.
12. Nieto-Castanon A. *Handbook of functional connectivity magnetic resonance imaging methods in CONN*. Hilbert Press; 2020.
13. Friston KJ, Williams S, Howard R, Frackowiak RS, Turner R. Movement-related effects in fMRI time-series. *Magnetic resonance in medicine*. 1996;35(3):346-355.
14. Hallquist MN, Hwang K, Luna B. The nuisance of nuisance regression: spectral misspecification in a common approach to resting-state fMRI preprocessing reintroduces noise and obscures functional connectivity. *Neuroimage*. 2013;82:208-225.
15. Nieto-Castanon A. Preparing fMRI Data for Statistical Analysis. *arXiv preprint arXiv:221013564*. 2022;
16. Yeo BT, Krienen FM, Sepulcre J*, et al*. The organization of the human cerebral cortex estimated by intrinsic functional connectivity. *Journal of neurophysiology*. 2011;
17. Sjuls GS, Specht K. Variability in resting-state functional magnetic resonance imaging: the effect of body mass, blood pressure, hematocrit, and glycated hemoglobin on hemodynamic and neuronal parameters. *Brain Connectivity*. 2022;12(10):870-882.
18. Hart A. Mann-Whitney test is not just a test of medians: differences in spread can be important. *Bmj*. Aug 18 2001;323(7309):391-3. doi:10.1136/bmj.323.7309.391
19. Wild C, Seber G. The Wilcoxon rank-sum test. *Chance Encounters: A First Course in Data Analysis and Inference*. 2011;611

20. Han H, Jiang X. Overcome support vector machine diagnosis overfitting. *Cancer Inform*. 2014;13(Suppl 1):145-58. doi:10.4137/cin.S13875
21. Wang C, Xiao Z, Wu J. Functional connectivity-based classification of autism and control using SVM-RFECV on rs-fMRI data. *Physica Medica*. 2019/09/01/ 2019;65:99-105. doi:https://doi.org/10.1016/j.ejmp.2019.08.010
22. Ding X, Yang Y, Stein EA, Ross TJ. Multivariate classification of smokers and nonsmokers using SVM-RFE on structural MRI images. *Human Brain Mapping*. 2015;36(12):4869-4879. doi:https://doi.org/10.1002/hbm.22956
23. Almairac F, Deverdun J, Cochereau J*, et al*. Homotopic redistribution of functional connectivity in insula-centered diffuse low-grade glioma. *Neuroimage Clin*. 2021;29:102571. doi:10.1016/j.nicl.2021.102571
24. Daniel AGS, Hacker CD, Lee JJ*, et al*. Homotopic functional connectivity disruptions in glioma patients are associated with tumor malignancy and overall survival. *Neurooncol Adv*. Jan-Dec 2021;3(1):vdab176. doi:10.1093/noajnl/vdab176
25. Liu D, Chen J, Hu X*, et al*. Contralesional homotopic functional plasticity in patients with temporal glioma. *Journal of Neurosurgery JNS*. 01 Feb. 2021 2021;134(2):417-425. doi:https://doi.org/10.3171/2019.11.JNS191982
26. Matthews BW. Comparison of the predicted and observed secondary structure of T4 phage lysozyme. *Biochimica et Biophysica Acta (BBA)-Protein Structure*. 1975;405(2):442-451.
27. Chicco D, Jurman G. The advantages of the Matthews correlation coefficient (MCC) over F1 score and accuracy in binary classification evaluation. *BMC genomics*. 2020;21:1-13.
28. Boughorbel S, Jarray F, El-Anbari M. Optimal classifier for imbalanced data using Matthews Correlation Coefficient metric. *PloS one*. 2017;12(6):e0177678.
29. Chicco D. Ten quick tips for machine learning in computational biology. *BioData mining*. 2017;10(1):35.
30. Barnard GA. A New Test for 2 × 2 Tables. *Nature*. 1945/12/01 1945;156(3974):783-784. doi:10.1038/156783b0
